# The *Streptococcus pyogenes* Rgg2/Rgg3 quorum sensing system causes global suppression of macrophage inflammatory programs via an intranuclear mechanism

**DOI:** 10.1128/mbio.00373-25

**Published:** 2025-09-25

**Authors:** Sam F. Feldstein, Kate M. Rahbari, Trevor R. Leonardo, Suzanne A. Alvernaz, Ian McIntire, Richard Foster, Michael J. Federle

**Affiliations:** 1 Department of Microbiology and Immunology, University of Illinois - Chicago 200799, Chicago, Illinois, USA; 2 Department of Biomedical Engineering, University of Illinois – Chicago 201650, Chicago, Illinois, USA; 3 Department of Pharmaceutical Sciences, University of Illinois – Chicago 315551, Chicago, Illinois, USA; University of Colorado Anschutz Medical Campus, Aurora, Colorado, USA

**Keywords:** group A Streptococcus, quorum sensing, immune evasion, innate immunity, macrophages, epigenetics

## Abstract

*Streptococcus pyogenes* or Group A Streptococcus (GAS), a significant human pathogen, employs quorum sensing (QS) systems to coordinate its behavior and genetic regulation to enhance survival. Our previous research established that one such QS system, the Rgg2/3 system, can suppress macrophage NFκB activity and production of pro-inflammatory cytokines. Yet, the scope of suppression and the mechanism by which it occurs remain unknown. In this study, transcriptomic and phosphoproteomic approaches were used to address these unanswered questions. It was found that QS-ON GAS broadly suppressed most inflammatory transcriptional pathways, including those of NFκB, type I and type II interferon responses, and intracellular stress responses. Yet, we found that no alternative transcriptional programs were activated after QS-ON GAS infection. Additionally, phosphoproteomics showed no disruption in typical inflammatory pathways such as those related to NFκB and MAPK activation, which was confirmed by western blotting and translocation assays. Instead, the proteomic data highlighted a potential role for epigenetic mechanisms of inflammatory regulation. A role for epigenetic regulation in QS-mediated immunomodulation was validated using pharmacological inhibitors of histone and chromatin modifiers. These findings expand our understanding of QS-mediated suppression and of GAS virulence strategies that appear to employ atypical methods of restricting inflammation. Uncovering this mechanism will offer invaluable insight into GAS itself, as well as into understudied immunological pathways.

**IMPORTANCE:**

*Streptococcus pyogenes* is a ubiquitous pathogen that causes over 600 million infections every year and 500,000 to 1 million fatalities. While in developed countries, it is generally known to cause mild conditions such as pharyngitis, it can also manifest as severe infections, such as necrotizing fasciitis and septic arthritis, and lead to post-infectious sequelae including rheumatic heart disease and glomerulonephritis. Elucidating new mechanisms of virulence in this organism, including how it evades and suppresses immune responses, is critical in understanding its pathogenicity and epidemiology, as well as identifying novel treatment avenues in this era of multidrug-resistant bacteria. In this study, we characterize the broad spectrum by which GAS modulates the host innate immune response and begin to uncover host pathways that the bacteria can use or inhibit for its survival.

## INTRODUCTION

*Streptococcus pyogenes*, also referred to as Group A Streptococcus (GAS), thrives as an ubiquitous human pathogen due to its extensive array of virulence factors. To maximize fitness and competitiveness inside the host, GAS employs various mechanisms to survive against the host immune system. One type of system that GAS can utilize is quorum sensing (QS), a regulatory system that enables coordinated genetic expression across a population of bacterial cells in response to microbially produced, intercellular signals. Other bacterial species, such as *Staphylococcus aureus and Pseudomonas aeruginosa*, also use their own QS systems to evade the immune system, often via the release of toxins (1, 2). In GAS, we previously identified and characterized the highly conserved Rgg2/Rgg3 QS system, which governs multiple mechanisms of protection against host defenses. These include lysozyme resistance, biofilm formation, and modulation of the host immune system (3–5).

The Rgg2/3 QS system consists of two transcriptional regulators, Rgg2 and Rgg3, which together coordinate activities to control genetic regulation of the system. Rgg2 functions as the transcriptional activator of the system, whereas Rgg3 is the transcriptional repressor (4). In the unstimulated state, Rgg3 predominates over the system and prevents transcription of regulated genes. Under certain conditions, the pheromone SHP (short hydrophobic peptide) can accumulate extracellularly. SHP peptides are imported into the cytosol, where they bind to both receptors Rgg2 and Rgg3. This interaction derepresses Rgg3 and activates Rgg2, driving transcription of a relatively small gene regulon (4, 6). Targets of the system include *shp* itself, thus incorporating a positive-feedback regulatory loop; *stcA*, which we have previously demonstrated promotes biofilm formation and confers the bacteria lysozyme resistance; and the 10-gene *spy49_0450-0460* operon, which we are in the process of characterizing (3, 5, 7). Given the distinct regulatory functions of Rgg2 and Rgg3, mutant strains lacking *rgg3* (*Δrgg3*) produce SHP pheromones constitutively and are therefore QS-active (referred to as “QS-ON”), while strains lacking *rgg2* (*Δrgg*2) cannot be stimulated and are thus QS-inactive (referred to as “QS-OFF”). Wild-type strains grown in a nutrient-rich chemically defined medium (CDM) are in a QS-inactive state due to rapid turnover of SHP, unless exogenous SHP is supplied (≥ 1 nM), which results in robust activation. We have found in mouse infection models of the upper respiratory tract or skin that the Rgg2/Rgg3 system is among the highest upregulated systems shortly after inoculation (8, 9).

We previously determined that QS-ON GAS inhibits macrophage NFκB transcriptional activity, as well as the production of cytokines including TNFɑ, IL-6, and IFNβ. This was understood to be an active suppressive phenotype given that mixed infections with both QS-ON and QS-OFF GAS at double the multiplicity of infection than either strain alone still induced a significantly attenuated response compared to QS-OFF alone. We further demonstrated that this suppressive phenotype is dependent on each of the first nine genes in the *spy49_0450-0460* operon (5). Ongoing work is aimed at characterizing the products and effects of this operon.

The ability of GAS to manipulate immune cell signaling has been incompletely characterized. GAS virulence factors have been most well studied for their ability to inhibit phagocytosis (10–12). A growing number of virulence factors have been shown to degrade host proteins, including immunoglobulins (13, 14), cytokines (15), and complement components (16). One of the most well-studied proteins is SpeB, a virulence factor regulated by the RopB QS system capable of cleaving both host and bacterial proteins (17–20). However, while many Gram-negative pathogens directly interfere with or suppress host immune signaling or activation, far fewer such virulence factors have been reported in Gram-positive bacteria (21, 22). *Staphylococcus aureus* secretes the protein extracellular fibrinogen-binding protein (Efb), which binds and disrupts TRAF3 in macrophages, leading to suppression of NFκB and AP1 signaling and the reduction of pro-inflammatory cytokine activation (23). An example that shares possible similarities to the suppression we observe in GAS is seen in *Mycobacterium tuberculosis*, which alters its mycolic acid structure, allowing it to bind the host receptor TREM2 to suppress the immune response (24, 25). In GAS, capsular hyaluronic acid has been shown to interact with inhibitory host receptors, leading to an attenuated immune response. However, our previous studies confirmed that the capsule was not necessary for QS-mediated suppression (5, 26).

Whereas our previous studies revealed that GAS utilizes QS signaling to suppress innate immune responses, the mechanism by which this occurs remains elusive. To address this gap, transcriptomic and proteomic approaches were utilized to globally examine differences in macrophage signaling networks after infection with QS-ON or QS-OFF GAS. We found that QS-ON GAS broadly suppresses transcription of inflammatory genes including those regulated by NFκB, interferons, and intracellular stress responses. Phosphoproteomic experiments showed that typical inflammatory signaling factors were not differentially activated, which was supported by western blots and analysis of transcription factor translocations. Rather, the results indicate that epigenetic or other intranuclear mechanisms cause the extensive transcriptional suppression observed. Uncovering this novel suppressive mechanism can help our understanding of the disease development and the severity of this ever-present pathogen.

## RESULTS

### QS-ON GAS globally suppresses macrophage inflammatory responses

**Fig 1:**
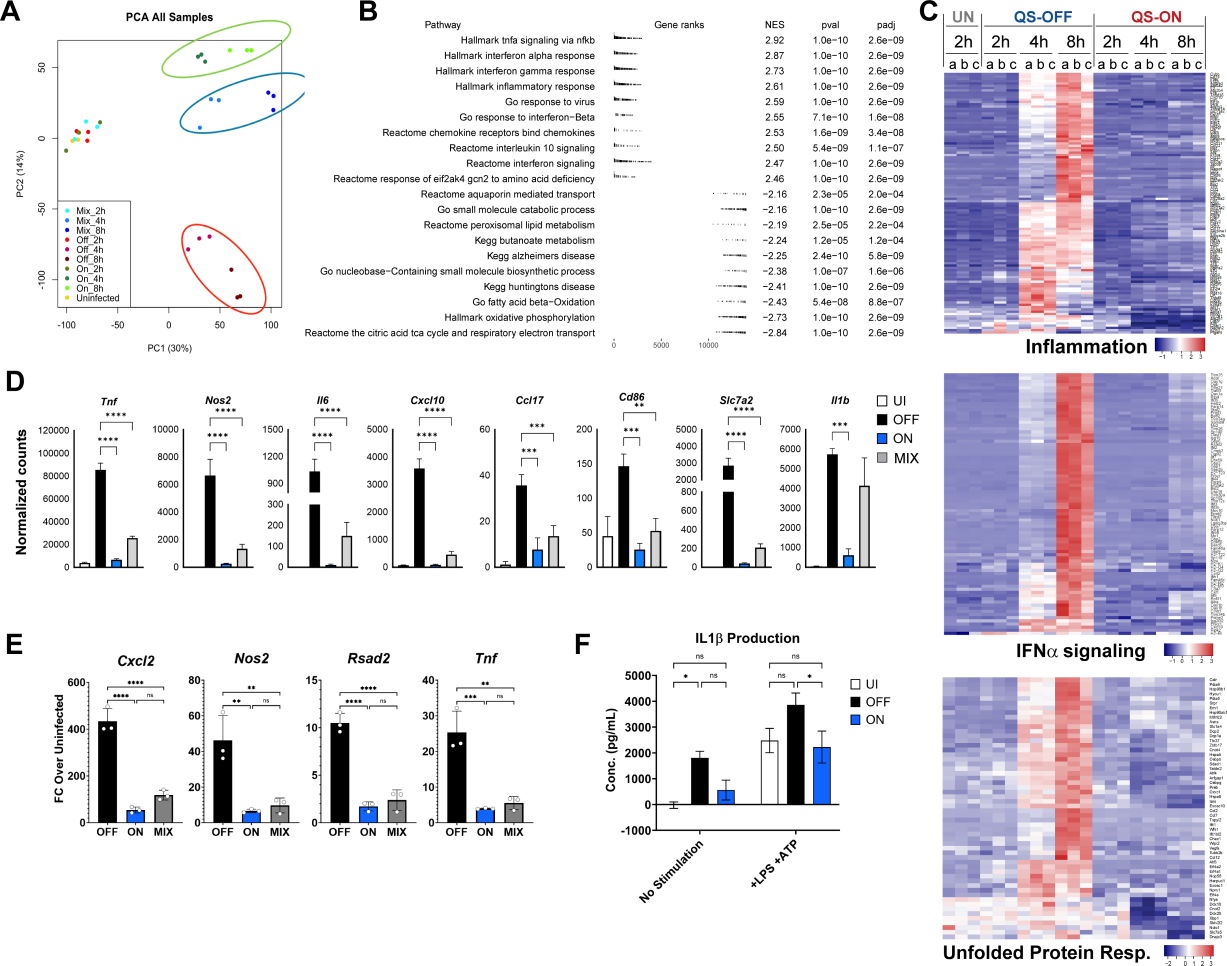
Global suppression of inflammatory genes by QS-ON GAS. (**A**) Principal component analysis showing individual RNA sequencing samples of uninfected and QS-OFF-, QS-ON-, and QS-MIX-infected RAW 264.7 cells at 2, 4, and 8 h post-infection (h.p.i.) with an MOI of 40. (**B**) Summary of gene set enrichment analysis showing the gene ranks and normalized enrichment scores (NES) of pathways in QS-OFF-infected cells relative to QS-ON. Highlighted are top upregulated and downregulated pathways from GSEA Hallmark, Reactome, GO, and KEGG gene sets. (**C**) Heatmaps comparing uninfected (UN), QS-OFF-, and QS-ON-infected cells at all time points from GSEA Hallmark gene sets of “Inflammatory_Response,” “IFNɑ_Response,” and “Unfolded_Protein_Response.” (**D**) Comparison of the normalized read counts at 8 h.p.i. time points from the RNA-seq study from select pro-inflammatory genes. (**E**) Validation of RNA-seq results of select genes by RT-qPCR at 4 h.p.i. with an MOI of 15 using J774.A macrophages. (**F**) IL-1β production from human-derived THP-1 cells infected with QS-OFF or QS-ON GAS. LPS was added 30 min p.i., ATP was added 3.5 h.p.i., and supernatant was collected 4 h.p.i. with an MOI of 20. All graphs show mean ± SEM. *P < 0.05; **P < 0.005; ***P < 0.001; ****P < 0.0001 by one-way ANOVA (**D and E**) or two-way ANOVA (**F**) with Tukey’s multiple comparison test.

To explore the scope and scale of the effect by which QS-ON Group A Streptococcus (GAS) suppresses inflammatory responses, we performed bulk RNA sequencing (RNAseq) of infected RAW 264.7 macrophages, collecting RNA from cells infected with Δ*rgg2* GAS (QS-OFF), Δ*rgg3* GAS (QS-ON), a 1:1 mixture of Δ*rgg2* + Δ*rgg3* GAS (QS-MIX), or from uninfected cells after 2, 4, and 8 h (Dataset S1). A total of 14,072 genes were identified, with 1,273 genes having an absolute-value difference between QS-ON and QS-OFF of 2-fold or greater and an adjusted *P*-value of at most 0.05 when averaged across all three time points. Principal component analysis confirmed that the three infection conditions formed distinct clusters at the 4- and 8-hour time points, with the QS-ON and QS-MIX clusters closer together than the QS-OFF cluster (Fig. 1A). It should be noted that the QS-ON and QS-MIX clusters were significantly separated from the uninfected and all 2-hour time points, indicating distinct transcriptional profiles from uninfected cells. We hypothesize that this is likely because the RNAseq data reveals that macrophages appear to still respond to QS-ON GAS, if still significantly less than to QS-OFF GAS.

To identify specific pathways that were differentially regulated after infection with QS-ON compared to QS-OFF GAS, Gene Set Enrichment Analysis (GSEA) was performed (27, 28). GSEA showed a reduced ability of macrophages to induce various inflammatory pathways, and in some cases even some anti-inflammatory pathways, when exposed to QS-ON GAS (Fig. 1B and C). These included pathways related to NFκB signaling, type I and type II interferon signaling, and IL-10 signaling. Inspection of individual typical inflammatory genes of interest, most of which are regulated by NFκB, confirmed that a large number and variety of inflammatory genes were suppressed in QS-ON- and QS-MIX-infected macrophages compared to QS-OFF-infected cells (Fig. 1D). The majority of these inflammatory genes were not significantly upregulated in the QS-OFF condition until 4 h post-infection. However, closer inspection of individual genes revealed that some were upregulated in QS-OFF-infected cells by 2 h and remained suppressed in the QS-ON infection, suggesting that suppression occurred early even if the overall effect size was not as strong. These early response genes include *Tnf*, *Cxcl2*, and *Atf3* (Fig. S1A). Suppression of several typical inflammatory genes of interest, such as *Cxcl2*, *Tnf*, *Rsad2*, and *Nos2*, which displayed among the largest fold changes between QS-OFF and QS-ON, was confirmed by RT-qPCR in J774.A and RAW 264.7 cells (Fig. 1E; Fig. S1D). Additionally, type I IFN-stimulated genes were found to be more suppressed compared to other inflammatory genes, a finding confirmed by RT-qPCR (Fig. S1C and E). Furthermore, inflammasome suppression by QS-ON GAS was confirmed via ELISA of secreted IL-1β in THP-1 cells (Fig. 1F). Together, these data indicate a widespread suppression of inflammation that likely impacts a variety of branches of inflammatory programs.

### QS-ON GAS does not induce an alternative transcriptional program in macrophages

**Fig 2:**
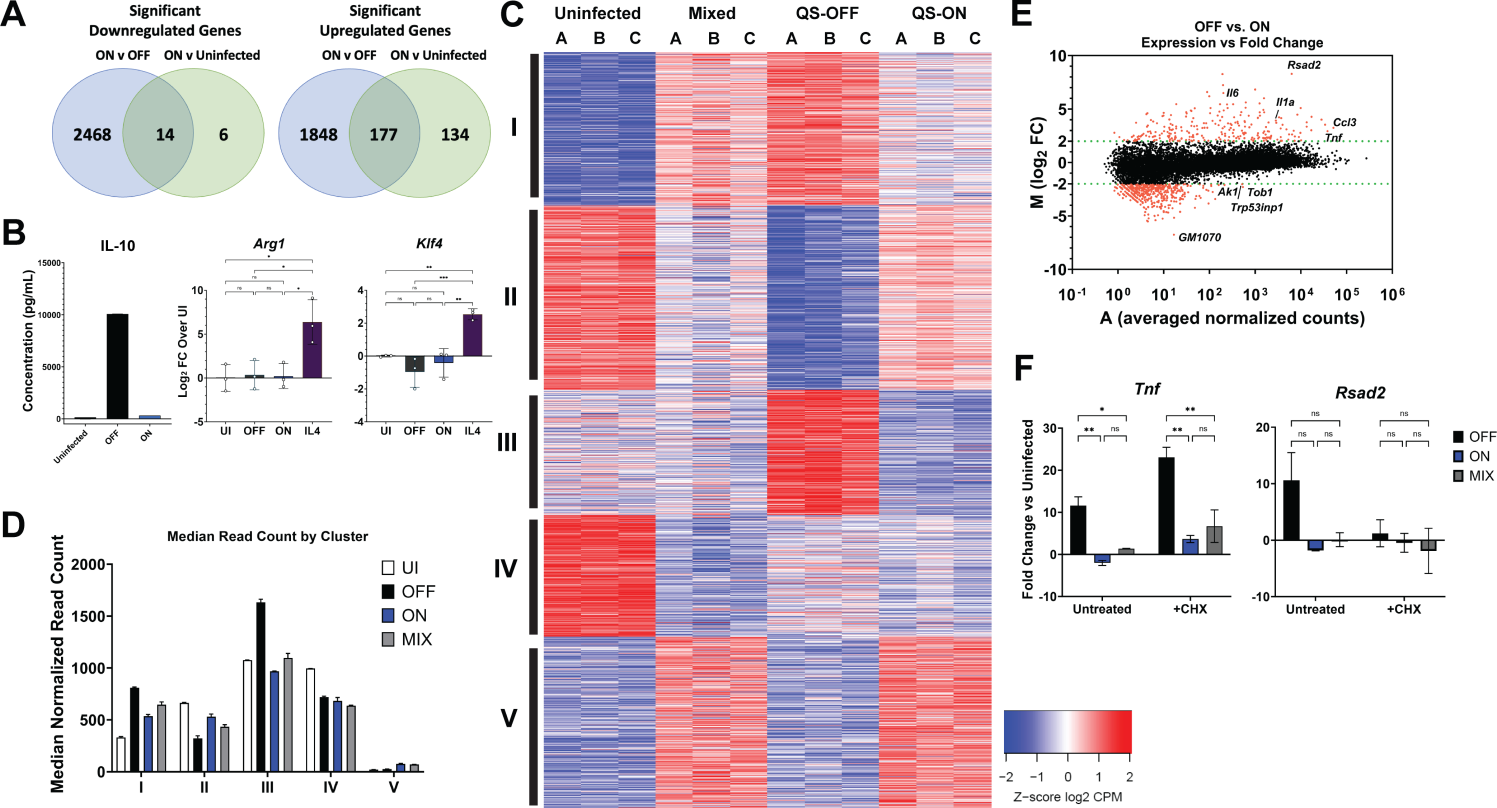
Differentially expressed gene clusters do not inform the mechanism of suppression. (**A**) Venn diagrams summarizing the number of genes that are commonly upregulated and downregulated between QS-ON-infected and QS-OFF-infected cells compared to those between QS-ON-infected and uninfected cells when averaged across all time points of the RNA-seq analysis. (**B**) IL-10 production in bone marrow-derived macrophage cells at 8 h post-infection (h.p.i.) measured by ELISA and representative of 2 separate experiments; gene expression of M2 macrophage markers *Arg1 and Klf4* in RAW 264.7 cells at 6 h.p.i. measured by RT-qPCR with an MOI of 15. IL4 was used as a positive control. (**C**) Clustering analysis of genes from RNA-seq that showed FDR-adjusted significance between any two conditions at 8 h. (**D**) Median normalized read count from RNA-seq analysis for each gene by cluster at 8 h. (**E**) MA plot showing the log_2_ fold change between QS-OFF- and QS-ON-infected cells (y-axis) and the average read count between both conditions averaged across all time points (x-axis). (**F**) Gene expression of *Tnf* (primary response gene) and *Rsad2* (secondary response gene) in J774 macrophages at 6 h.p.i. with an MOI of 20 in the presence or absence of the translation inhibitor cycloheximide (CHX) measured by RT-qPCR. 10 µM CHX was added 15 min prior to infection and remained until cell collection. Graphs show mean + SEM. *P < 0.05; **P < 0.005 by ANOVA (**E and F**) with Tukey’s multiple comparison test.

Besides showing downregulation of inflammatory pathways in QS-ON-infected macrophages, GSEA analysis also indicated that QS-ON-infected macrophages exhibited higher expression of genes in pathways related to typical alternatively activated (M2) macrophages, such as fatty acid beta-oxidation and oxidative phosphorylation (Fig. 1B). However, comparing the fold changes of the genes in these pathways between infected and uninfected cells, the differences are best described as being due to a greater downregulation of these pathways in the QS-OFF-infected condition rather than due to their stimulation by QS-ON bacteria. In fact, of the 2,025 genes more highly expressed in QS-ON- versus QS-OFF-infected cells, only 177 genes were also elevated compared to the uninfected control (Fig. 2A). However, these 177 genes are not related by a known common pathway or mechanism by gene ontology (GO) analysis. Still, because the induction of M2-associated cytokine secretion by microbes has been described as a common mechanism to suppress inflammatory responses (29, 30), we tested levels of secreted IL-10, an immunosuppressive cytokine, across infection conditions. Consistent with the RNAseq data, IL-10 secretion was highest in the QS-OFF-infected cells (Fig. 2B; Fig. S1B), which is consistent with previous research showing that IL-10 is type I IFN-dependent in GAS infections (31). This negates the likelihood that M2 polarization accounts for the observed QS-ON-dependent suppression and further supports that QS-ON broadly suppresses all types of transcriptional programs rather than inducing an alternative program. Likewise, common genes associated with alternatively activated macrophages, such as *Arg1* and *Klf4*, were not upregulated in QS-ON-infected macrophages when measured by RT-qPCR (Fig. 2B).

Despite QS-ON GAS not inducing M2 polarization, we considered that it still may induce other pathways or programs. To observe relative transcription patterns across all infection conditions, a k-means clustering analysis of all differentially expressed genes at the 8-hour time point was performed, which revealed five distinct clusters (Fig. 2C). Genes in Cluster I correspond to most of the inflammatory genes that are less strongly upregulated after infection with QS-ON or QS-MIX GAS compared with QS-OFF GAS (Fig. S2A). Clusters II–IV also map well to known biological functions such as those related to metabolism, chromatin regulation, and cell cycling (Fig. S2B through D). However, Cluster V, which contains genes uniquely upregulated in both the QS-ON and QS-MIX conditions (Table S2), and which would therefore most likely inform on a mechanism controlled by QS-ON GAS, did not map to any GO terms. Additionally, as the overall median expression values of genes in cluster V are substantially lower than in the other groups, we suspect that these differences may not be biologically relevant (Fig. 2D). To determine if the expression levels of any of the genes upregulated in the QS-ON condition compared to QS-OFF appeared biologically relevant, we plotted all the measured genes on an MA plot, which compares the fold change between OFF vs. ON (y-axis) and mean expression value between the two conditions (x-axis) (Fig. 2E). This revealed that genes upregulated in the QS-OFF condition span a wide range of expression levels, while those upregulated in the QS-ON condition are clustered toward very low expression levels, with many showing a normalized read count below 10. Thus, whereas changes in transcriptional profiles are striking between QS-ON and QS-OFF infections, they do not reveal a clear mechanism for suppression or activation of an alternative transcriptional program. Finally, when macrophages were treated with the translation inhibitor cycloheximide (CHX), QS-ON- and QS-MIX-infected macrophages still exhibited reduced expression of inflammatory genes, indicating that suppression is not caused by any newly transcribed protein induced by QS-ON GAS interaction with the cell (Fig. 2F). We conclude that QS-ON GAS does not induce an alternative program of transcription, largely only suppress transcription rather than significantly inducing a large number of genes, and that suppression is not dependent on induction of a newly synthesized protein.

### Phosphoproteome of infected cells suggests an epigenetic mechanism of suppression by QS-ON GAS

**Fig 3:**
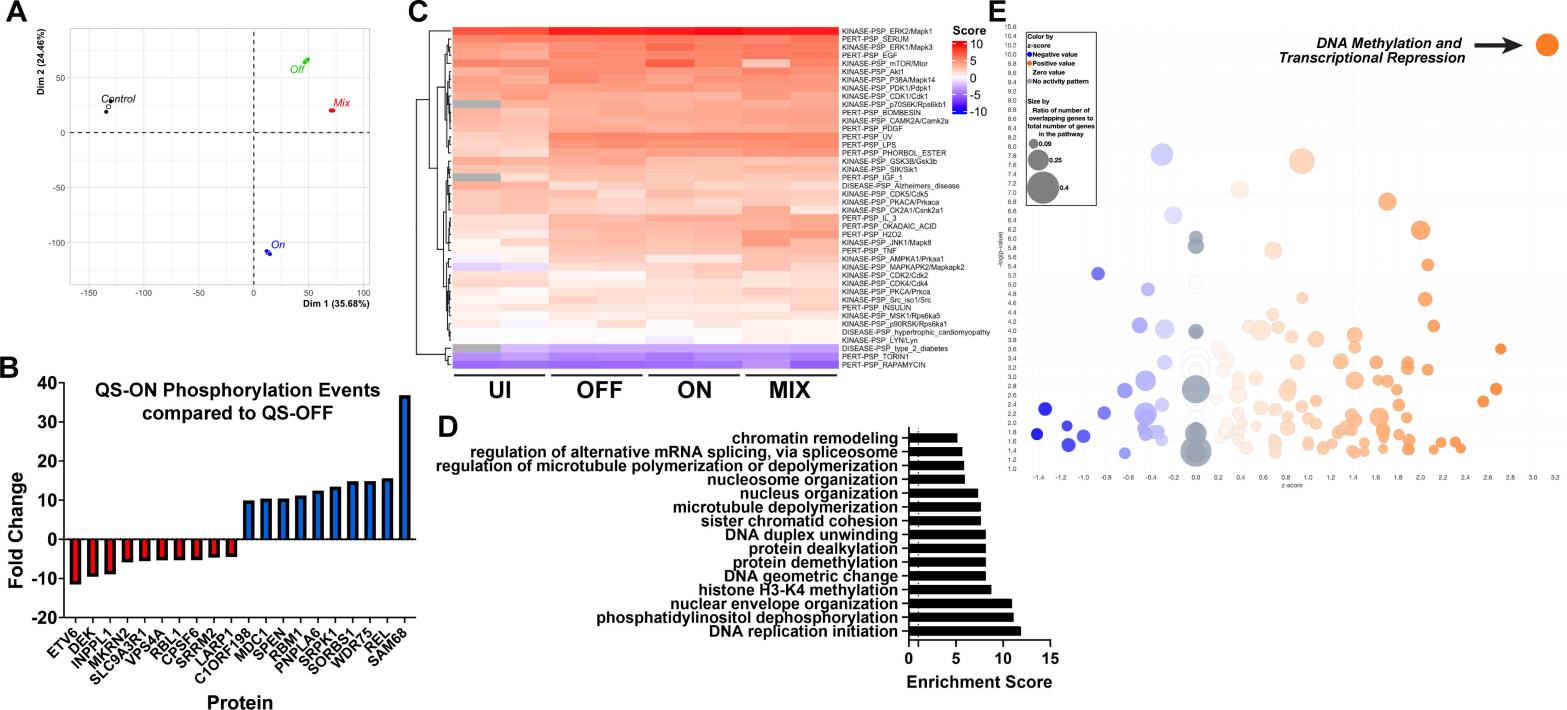
PTM scan reveals possible epigenetic mechanism of suppression. (**A**) Principal component analysis of PTM scan, measuring the abundances of phosphorylated proteins in uninfected or QS-ON-, QS-OFF-, and QS-MIX-infected RAW 264.7 cells 30 min post-infection (p.i.) with an MOI of 40. (**B**) Proteins with the greatest differential abundance observed by post-translational modification (PTM) analysis. (**C**) Heatmap of PTM-Signature Enrichment Analysis (PTM-SEA) showing relative enrichment scores of each sample (uninfected, OFF, ON, or MIX conditions) for each annotated pathway. (**D**) Gene ontology analysis of PTM scan showing the highest enriched pathways upregulated in QS-ON-infected cells compared to QS-OFF-infected. (**E**) Ingenuity Pathway Analysis (IPA) of PTM scan depicting its predicted upregulated (orange) and downregulated (blue) pathways in QS-ON-infected cells compared to QS-OFF-infected. DNA methylation and transcriptional repression pathway is indicated by the arrow in the top right corner of the graph.

Because transcriptomics revealed global immunosuppression rather than alteration of a specific pathway, we hypothesized that QS-ON GAS might alter inflammatory signaling pathways, which are generally transduced via protein phosphorylation. To test this, we performed a post-translational modification (PTM) scan examining all detectable phosphorylated proteins 30 min post-infection of RAW 264.7 macrophages with QS-OFF, QS-ON, and QS-MIX, compared to steady-state levels of uninfected cells (Dataset S2). We detected 20,572 phosphorylation events across 3,393 different proteins. Principal component analysis revealed distinct clusters for the QS-OFF, QS-ON, and QS-MIX conditions (Fig. 3A). Interestingly, the QS-ON infected condition clustered furthest from the uninfected condition. Whereas the RNAseq data showed that QS-ON and QS-MIX conditions were more similar to each other, the PTM data showed that QS-OFF and QS-MIX conditions were more similar.

To find pathways differentially activated or suppressed between infection conditions via phosphorylation, PTM-Signature Enrichment Analysis (PTM-SEA) was performed using both mouse and human signature databases (human data not shown) (32). Enrichment scores for the PTM-SEA pathways revealed no significant differences between the QS-ON, QS-OFF, and QS-MIX conditions in classical inflammatory or other pathways (Fig. 3B and C; Fig. S4A and B). Classical inflammatory pathways and proteins, such as those related to TNFɑ, NFκB, and MAPK signaling, displayed low levels of phosphorylation in the uninfected condition as expected, while all infected conditions showed similar levels of phosphorylation of proteins in these pathways. Other pathways, including those related to CDK2 and GSK3β, showed increased and decreased activation, respectively, between QS-ON-infected and QS-OFF-infected cells and between QS-MIX-infected and QS-OFF-infected cells (Fig. S4B). When performing GO enrichment analysis, comparing phosphorylation levels of proteins in QS-ON- vs. QS-OFF-infected macrophages, several pathways related to epigenetic regulation, such as “histone H3-K4 methylation” and “protein de-acetylation,” were enriched (Fig. 3D). This finding was supported by Ingenuity Pathway Analysis (IPA), in which “DNA Methylation and Transcriptional Repression” was the highest and most significantly upregulated predicted pathway in the QS-ON vs. QS-OFF conditions (Fig. 3E).

**Fig 4:**
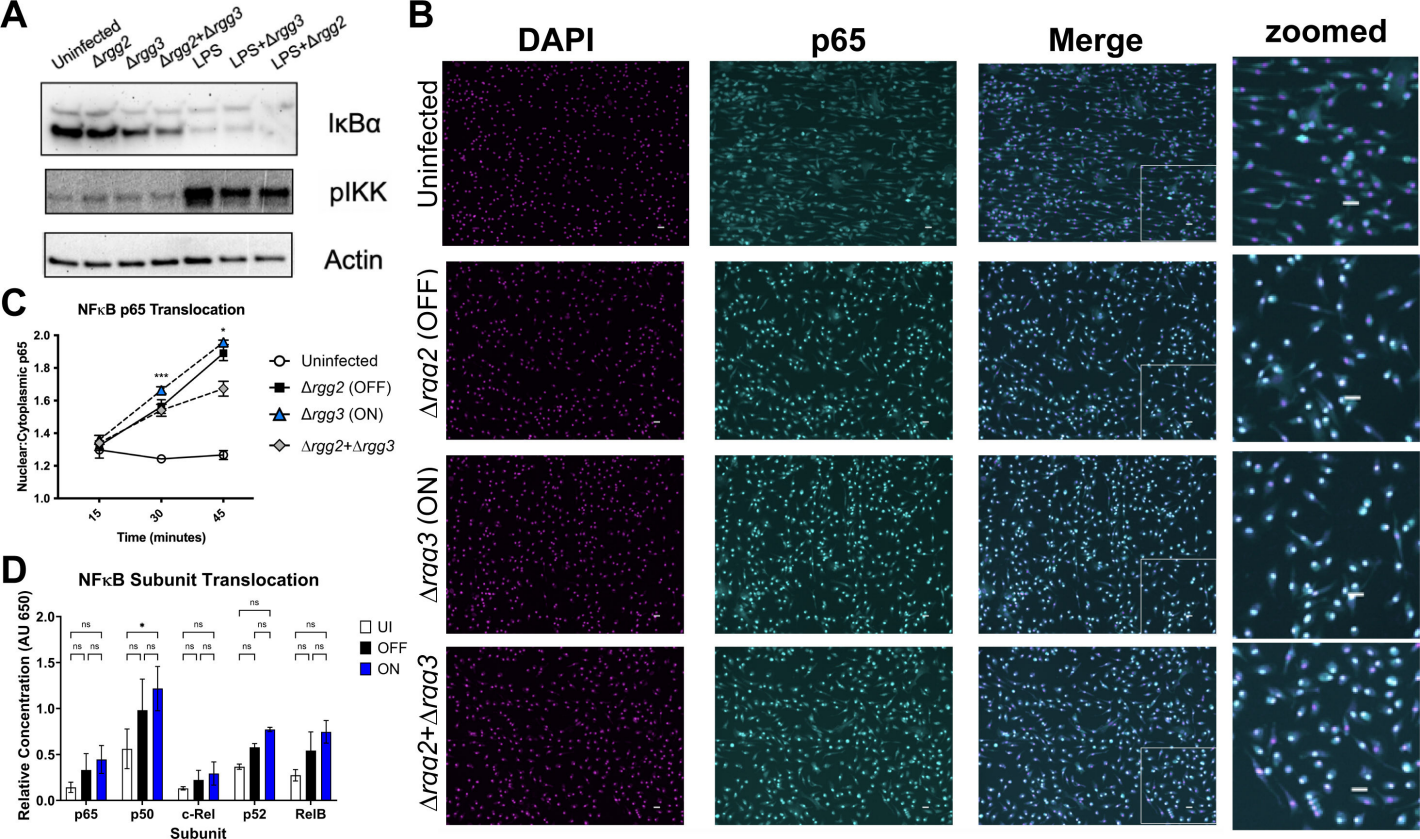
Translocation of NFκB is unaltered between QS-OFF- and QS-ON-infected macrophages. (**A**) Representative western blots of NFκB pathway components IκBɑ and phospho-IKK at 30 min post-infection (p.i.) with an MOI of 40. (**B**) Representative images of immunofluorescence microscopy staining in BMDMs for DAPI (magenta), and p65 (cyan) 45 min p.i. with an MOI of 50. The right column is a zoomed box shown in the merged fluorescence images. Scale bar, 20 mm. (**C**) Quantification of p65 translocation to the nucleus measured by microscopy. Shown is the mean ratio of mean fluorescence intensity of nuclei compared to cytoplasm as quantified using CellProfiler. Ratios from five fields were averaged. Graph shows mean + SD. (**D**) Relative nuclear abundance of each NFκB subunit in differentiated THP-1 cells at 35 min. p.i. with MOI of 20 measured with TransAM NFκB Family Kit (Active Motif). The graph shows mean ± SEM.

Because inflammatory signaling proteins were not differentially phosphorylated, while epigenetic pathways appeared enriched in QS-ON-infected macrophages, we hypothesized that epigenetic or transcriptional regulation within the nucleus may be the point of cellular activation that is impacted by QS-ON-infected cells. To confirm that upstream NFκB activation was not altered between infection conditions, IKK phosphorylation and IkB degradation were evaluated. QS-ON- and QS-MIX-infected macrophages showed similar IKK phosphorylation and higher or equal IkB degradation than QS-OFF-infected macrophages (Fig. 4A). Similar results were observed with or without co-stimulation with LPS, a well-studied TLR agonist, and our previous work showed that NFκB-dependent transcriptional responses induced by LPS and other TLR agonists are suppressed by QS-ON GAS (5). Immunofluorescence microscopy also confirmed that translocation of p65, a typical pro-inflammatory subunit of NFκB, translocates to the nucleus in QS-ON-infected cells as well as in those infected with QS-OFF (Fig. 4B and C). In fact, this trend is consistent among all five NFκB subunits, as determined by an ELISA-based assay (Active Motif) that uses the consensus binding sequence to capture the substrate (Fig. 4D). These data support the hypothesis that suppression is not occurring at upstream signaling points, but rather within the nucleus after translocation.

### QS-ON-dependent suppression relies on epigenetic regulation

**Fig 5:**
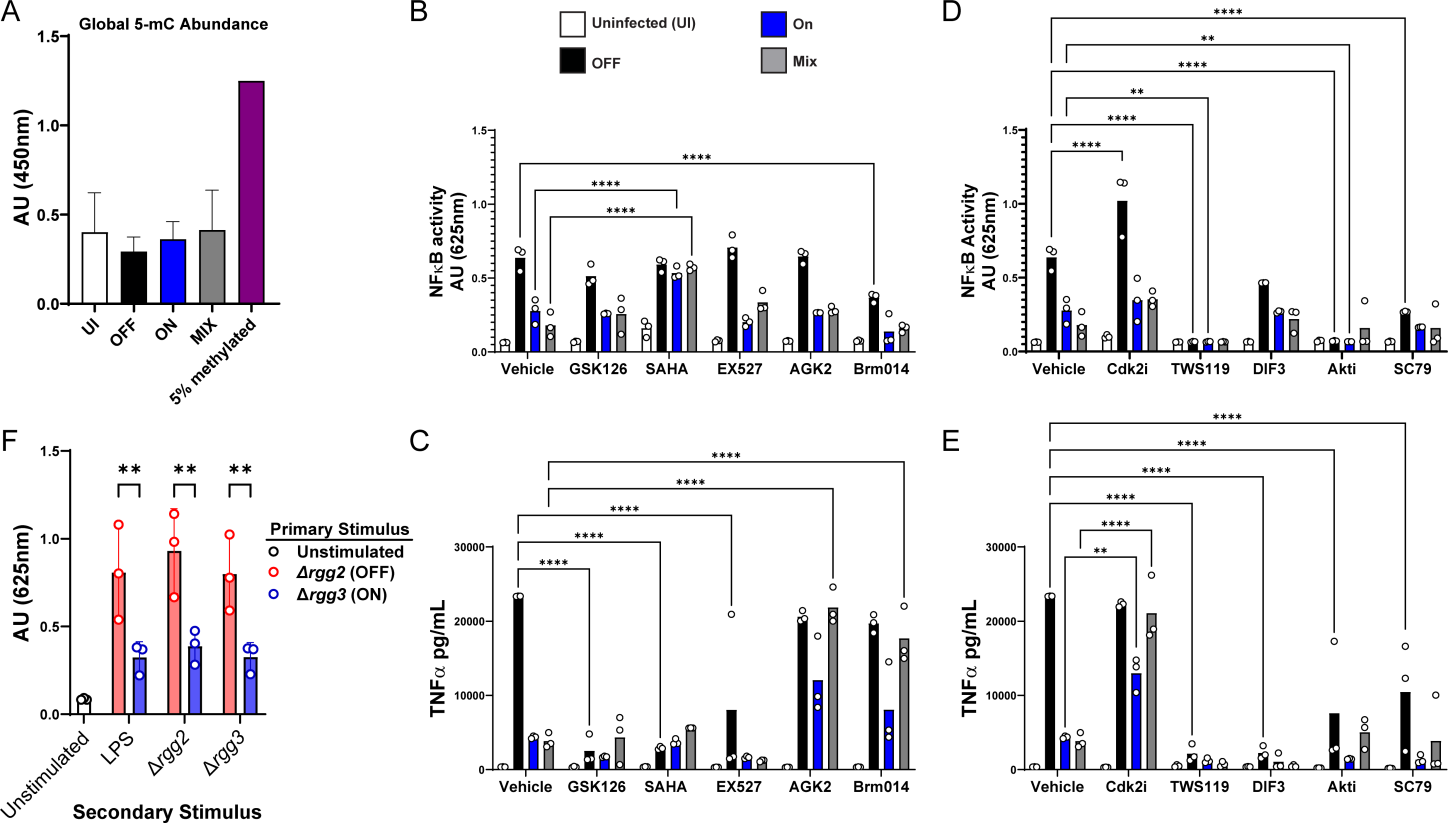
Pharmacological inhibition of epigenetic regulators does not restore inflammatory capacity of macrophages. (**A**) Quantification of global 5-methylcytosine abundance in RAW 264.7 cells 3 h post-infection (h.p.i.) with indicated GAS strains, measured with Epigentek MethylFlash kit (*n* = 2). (**B and C**) NFκB reporter activities (**B**) or TNFa amounts (**C**) in RAW-Blue cells 18 h.p.i. with the indicated GAS strains treated with DMSO vehicle or 5 µM GSK126, 500 nM SAHA, 10 µM EX527, 5 µM AGK2, or 1 µM BRM014. (**D and E**) NFκB reporter activities (**D**) or TNFa amounts (**E**) in RAW-Blue cells 18 h.p.i. with indicated GAS strains treated with DMSO vehicle or 2 µM CDK2 inhibitor II, 7.5 µM TWS119, 7.5 µM DIF-3, 20 µM AKT1/2 inhibitor, or 10 µM SC79. (**F**) NFκB activation of RAW-Blue cells challenged with indicated GAS strains for 30 min (primary stimulus), then recovered in 100 ug/mL gentamycin for 20 h, washed, and reinfected with indicated GAS strain or 100 ng/mL of LPS (secondary stimulus). Panels **B–F** are results of three biological replicates; **P < 0.005, ****P < 0.0001 by ANOVA with Tukey’s multiple comparison test.

Given that Ingenuity Pathway Analysis of the PTM data predicted “DNA Methylation and Transcriptional Repression” as the strongest predicted upregulated pathway in QS-ON vs QS-OFF-infected cells, we first examined global DNA methylation levels in infected cells using an ELISA that measures 5-methylcytosine (5-mC). Given the global suppression observed in the RNAseq data, we hypothesized that if DNA methylation was the mechanism of suppression, it would be measurably evident in such an assay. However, no differences in DNA methylation levels were observed across any of the infection conditions or in the uninfected control (Fig. 5A).

Next, to test whether alternative regulation of histones was a mechanism of suppression, several selective inhibitors of histone modifiers were utilized to observe if they blocked the suppression of QS-ON GAS measured by NFκB activity and production of the inflammatory cytokine TNFɑ. We initially looked at common histone modifiers that also appeared as differentially phosphorylated in the PTM scan, such as EZH2, HDAC1, and HDAC2 (Fig. S4C and D). GSK126 was used to inhibit EZH2, the key catalytic protein of the polycomb repressive complex 2 (PRC2), which has been shown to be involved in inflammatory regulation (33, 34). Suberoylanilide hydroxamic acid (SAHA) is a class I, II, and IV histone deacetylase (HDAC) inhibitor and has been shown to have anti-inflammatory properties (35, 36). Other epigenetic regulators with prior evidence of roles in inflammatory involvement were also investigated. EX-527 and AGK2 were used as sirtuin 1 and sirtuin 2 inhibitors, respectively. Sirtuins are class III HDACs, which have been shown to be targets of bacterial host immune regulation strategies (37, 38). Lastly, BRM014 was used to inhibit BRG1, a key component of the SWI/SNF complex, which is involved in chromatin remodeling and shown to be involved in inflammatory processes (39). Application of SAHA had the expected result of reducing production of TNFa, most substantially in the QS-OFF condition, but was also seen to eliminate the suppressive impact of QS-ON in the NFκB reporter (Fig. 5B and C). GSK126 and EX527 also lowered TNFa levels, but their impact on QS-ON-dependent suppression was not evident at the level of NFκB. AGK2 and BRM014 did not lower TNFa in the QS-OFF infected cells, although suppression by QS-ON bacteria was slightly diminished.

We also investigated whether CDK2 or GSK3β pathways were potentially involved in suppression, given their presence in the PTM-SEA phosphoproteome pathway analysis (Fig. S4B). They have been shown to be involved in both inflammatory and epigenetic regulation (40–44). CDK2 Inhibitor II (CDK2i) was used to investigate CDK2, while DIF-3 and TWS119 were used to induce and inhibit GSK3β, respectively. Additionally, because GSK3β is closely tied with the AKT pathway (45), SC79 was used to induce AKT, while Akt1/2 kinase inhibitor (AKTi) was used to inhibit AKT. The CDK2 inhibitor had a modest effect on elevating levels of TNFa in the QS-ON and MIX conditions, but this loss of suppression was not seen at the level of NFκB (Fig. 5D and E). Inhibitors of GSK3b and Akt were seen to have an overall suppressive effect on TNFa production and NFκB activities, but the inducer of both proteins also had strong negative effects. Thus, it is difficult to ascertain the roles of these proteins in QS-dependent suppression except to report that their dysregulation impacts inflammatory responses to GAS.

As results from histone-modification inhibitors provided the strongest link to QS-dependent suppression, we reasoned that if QS-ON GAS were inducing an epigenetic change, suppression should remain evident after reinfection, similar to what has been described in epithelial cells infected with *Streptococcus pneumoniae* (45–47). Therefore, we carried out macrophage infections in two stages: first, infecting cells with either QS-ON or QS-OFF bacteria for 30 min, followed by incubation in antibiotics for 20 h. Macrophages were subsequently washed with antibiotic-free media and re-infected with either QS-ON or QS-OFF bacteria or treated with the TLR4 agonist LPS. Twenty hours after the secondary infection or treatment, NFκB activity was measured. We found that macrophages exposed to QS-ON bacteria in the primary event were impaired in their ability to induce inflammatory responses to the secondary treatment, regardless of the stimulus (Fig. 5F). In contrast, macrophages infected with QS-OFF bacteria during the primary exposure were able to stimulate a higher NFκB response. These results are consistent with the idea that epigenetic regulation of gene expression contributes to the suppressive effects elicited by QS-ON GAS. Interestingly, macrophages treated with QS-OFF in the primary infection could not be suppressed by QS-ON GAS in the secondary exposure. Therefore, once conditioned, macrophages retained the activation potential set by the initial encounter with GAS in the respective QS state.

## DISCUSSION

These results show that quorum-sensing–ON Group A Streptococcus (QS-ON GAS) has a global inhibitory effect on inflammatory responses in macrophages, reducing the potency of most, if not all, inflammatory programs. Not only is inflammation significantly attenuated, but no other pathways were seen to be triggered by QS-ON GAS that would account for macrophage inactivation. This is consistent with our findings that suppression does not occur at the point of upstream signaling, but rather after transcription factors have translocated to the nucleus, affecting transcription directly. A post-translational modification (PTM) scan analyzing the phosphoproteome suggests that the suppression is occurring via epigenetic regulation of inflammatory transcription.

Although our previous study characterized the phenomenon of QS-ON as having the ability to actively suppress the TLR-NFκB axis of inflammatory responses—hallmarked by a reduction of key inflammatory markers (i.e., IL-6, TNFɑ, IFNβ)—the level of signaling at which activation was blocked and the scope of suppression were unknown. Additionally, the mechanism of how the suppression occurred was unknown. Here, we expand upon that work by showing the full scale of suppression and demonstrating that it occurs after transcription factors have translocated to the nucleus. Although we anticipated that QS-ON GAS would prevent activation of NFκB, we saw no differences in phosphorylation of key signaling pathways leading to activation of NFκB or MAPK/AP1, and all NFκB subunits translocated to the nucleus at similar rates. Instead, PTM analysis indicated that epigenetic regulation is a possible mode of suppression, which explains why signal transduction pathways promoting inflammation were not altered by QS-ON GAS interaction.

RNAseq data sets did reveal some unanticipated responses to GAS infections. One unexpected pattern was observed in classical type I interferon (IFN)–stimulated genes (ISGs). While classical pro-inflammatory genes, such as *tnf*, *nos2*, *il6*, and others, exhibit significantly higher expression in QS-OFF vs. QS-ON conditions, they also show significantly higher expression in QS-ON vs. uninfected conditions. Thus, the effect of the Rgg2/Rgg3 QS system could be best described as an attenuation of inflammation rather than complete inhibition. However, this is not what is observed with regard to type I ISGs, where complete or near-complete inhibition is observed for these genes in QS-ON-infected cells. Classical type I ISGs, such as *rsad2*, *mx2*, and the *ifit* genes, all have minimal to no upregulation in the QS-ON condition compared to uninfected. We hypothesize that this occurs because the production of type I IFNs is significantly reduced in QS-ON-infected cells, which we have previously shown by ELISA, and therefore does not reach a sufficient critical concentration to induce production of these ISGs. This may indicate the ultimate benefit received by the bacteria when they activate quorum sensing. While an incomplete reduction in primary response proteins may aid in the survival and persistence of the bacteria, it may be the elimination of the type I IFN response that creates the necessary environment the bacteria require. Furthermore, if type I IFN secretion does not reach sufficient concentrations to trigger paracrine signaling *in vitro*, it seems likely that other attenuated cytokines that communicate to different cells via paracrine or even endocrine signaling would also not be in sufficient concentrations to enact their effects. The effects of type I IFN on GAS fitness and assays measuring cell-to-cell signaling, such as recruitment assays in the context of QS-ON GAS, would be ideal future experiments to perform.

While we have shown that activation of typical inflammatory pathways such as those of NFκB and AP1 does not appear to be altered in a QS-ON infection, we are limited by the scope of our post-translational medication scan which only measured phosphorylated proteins. It is likely that we only see minimal differences in pathways between QS-ON- and QS-OFF-infected macrophages because the signaling is being transduced via alternative modifications. Several inflammatory signaling pathways are induced via signals and switches other than phosphorylation, including GTP, cAMP, SUMOylation, ubiquitination, hydroxylation, and others (48–52). However, many of these signals also include a phosphorylation step (such as the cAMP-PKA axis), which was not observed in our PTM scan.

Additionally, while our data strongly suggests an intranuclear mode of inflammatory suppression, we have yet to uncover the particular mechanism. Still, we have confidently ruled out the mechanism being inhibition of upstream signaling molecules in the NFκB and likely AP1 pathways. With these pathways unaltered even up to the point of translocation into the nucleus, the site of inhibition must be within the nucleus. While the epigenetic regulators we investigated did appear to be involved in QS-mediated suppression, we still have not ruled out completely the involvement of another epigenetic protein. These include proteins such as ARID4B, which shows significantly higher phosphorylation in the QS-ON- and QS-MIX-infected macrophages. Forthcoming work aims to look at these proteins and regulators, as well as to directly assess histone modifications and chromatin accessibility. Additionally, we have not ruled out the potential role of other intranuclear regulators, such as co-activators of transcription, other components necessary for the transcription machinery, or noncoding RNAs. We have equally not eliminated the possibility of post-transcriptional mRNA degradation, a mode of regulation used by pathogens and the host alike (53, 54). The possibility of more broad transcriptional suppression not specific to inflammatory processes is evidenced by the downregulation of other pathways such as the unfolded protein response in the RNAseq data set. Lastly, while we have shown that QS-ON GAS does not directly promote metabolic pathways transcriptionally, the remaining activity or inactivity of these pathways may also contribute to the observed suppressive phenotype, as others have shown that certain metabolic processes may be required to maintain or drive inflammatory states (55–57). Future work will further investigate the mentioned alternative modes of signal transduction, possible alternative mechanisms of transcriptional regulation within the nucleus, and finally immunometabolism.

## MATERIALS AND METHODS

### Bacterial strains

*Streptococcus pyogenes* (Group A Strep, GAS) NZ131 strain was used for all experiments. Δ*rgg2 and Δrgg3* strains were constructed as previously described (4). Starter cultures were used to minimize differences in lag phase between strains and experiments and were created as previously described (5).

### Cell lines

RAW 264.7 murine monocyte/macrophages were cultured in RPMI 1640 (Corning) supplemented with 10% fetal bovine serum (FBS) (BenchMark) and penicillin/streptomycin (P/S) (Corning).

THP-1 human monocytes were cultured in identical complete RPMI 1640 media. To differentiate the monocytes into M0 macrophages, cells were treated with 5 ng/mL of phorbol 12-myristate 13-acetate (PMA) in complete media for 48 h, after which the cells were washed with PBS and incubated with antibiotic-free and PMA-free media for 24 h.

J774A.1 murine macrophages were cultured in DMEM (Gibco) supplemented with 10% FBS and P/S. RAW-Blue cells (Invivogen), which contain an intrachromosomal insertion of the secreted embryonic alkaline phosphatase gene driven by both NFκB- and AP1-reactive promoters, were cultured in identical complete DMEM with the addition of 200 µg/mL of Zeocin every other passage to maintain selection pressure.

### Generation of bone marrow-derived macrophages

Primary murine bone marrow–derived macrophages (BMDMs) were differentiated from bone marrow (BM) cells collected from the femurs and tibiae of six- to eight-week-old male or female C57BL/6 mice (Charles River Laboratories). BM cells were frozen and differentiated at a later date as previously described (5).

### *In vitro* infections

Designated cell lines were seeded into tissue culture-treated plates (Falcon) in antibiotic-free media and allowed to settle overnight. The following day, GAS strains were grown from frozen starter cultures in CDM to an OD_600_ of 0.5–0.6. The separate strains were normalized by their OD, centrifuged, resuspended in the cell culture medium, and then added to the macrophages. The cells were centrifuged at 300 × *g* for 5 min to synchronize bacterial contact with the cells and then incubated for 30 min at 37°C with 5% CO_2_ , unless otherwise noted. After incubation, the wells were washed and replaced with new media containing 100 µg/mL of gentamycin (Gibco) to kill any remaining extracellular bacteria.

### RNA sequencing

RAW 264.7 cells were prepared in RPMI 1640 and infected as described above using a multiplicity of infection (MOI) of 40 CFUs per cell. Cells were infected with Δ*rgg2*, Δ*rgg3*, an MOI of 20 each of Δ*rgg2* + Δ*rgg3*, or bacteria-free media. Infected cells were collected at 2, 4, and 8 h post-infection, while the uninfected cells were only collected at 2 h post-infection. At the appropriate time point, cells were collected and stored in RNAlater (Invitrogen) at −80°C. Cells were later thawed, and their RNA was extracted using RNeasy Mini Kit (Qiagen) and RNase-Free DNase Set (Qiagen) and sent to Novogene for library preparation and sequencing.

At Novogene, RNA was quality control tested via Nanodrop for quantitation, gel electrophoresis for degradation and contamination, and Agilent 2100 Bioanalyzer for integrity and quantitation. mRNA was enriched using oligo(dT) beads, and rRNA was depleted using Ribo-Zero kit (Illumina). mRNA was randomly fragmented using fragmentation buffer, and cDNA was synthesized by second-strand synthesis using random primers and a custom second-strand synthesis buffer (Illumina). The cDNA was end-repaired, followed by 3′ polyadenylation and sequencing adaptor ligation. The library was then size selected and enriched by PCR. Quality control was performed on the cDNA library via Qubit 2.0 (Thermo) for concentration, Agilent 2100 Bioanalyzer for insert size, and qPCR for precise concentration. Qualified libraries were pooled and sequenced paired-end at 2 × 150 bp (Illumina).

### RNAseq analysis

Data quality control was performed on FASTQ files using FASTQC, and the raw sequencing reads were aligned to the mouse genome (GRCm39) using STAR. Raw sequencing reads of genomic features were enumerated using featureCounts, and DESeq2 was used to normalize the counts, provide differential expression analysis, and generate pre-ranked test-statistic data. fGSEA algorithm with mouse MSIGDB was used to perform a functional gene set enrichment analysis.

Separately, the featureCounts of uninfected and 8-hour time point samples were normalized and analyzed using edgeR. Genes were then filtered based on whether they have an FDR-adjusted *P*-value < 0.05 for at least one pairwise sample comparison. The resulting genes were clustered by k-means clustering, with clustering performed 10 times for a *k* varying from 2 to 20. *k* = 5 was chosen by highest reproducibility.

### RT-qPCR

For RNA-seq validation and M2 gene investigation, RAW 264.7 cells were prepared in RPMI 1640 and infected as described above using an MOI of 10. Cells were infected with Δ*rgg2*, Δ*rgg3*, or Δ*rgg2* + Δ*rgg3*, or bacteria-free media or stimulated with 100 ng/mL of LPS (Sigma) or 20 ng/mL of recombinant murine IL-4 (Sigma).

For translation inhibition assays, J774A.1 cells were used. 10 µg/mL of cycloheximide (Sigma) was added 20 min before the infection and remained until the cells were harvested.

Cells were collected at 0, 4, 6, and/or 8 h post-infection, and RNA was collected using RNeasy Mini Kit (Qiagen). cDNA conversion was performed using High-Capacity cDNA Reverse Transcription Kit (Applied Biosystems), and qPCR was performed using PowerUp SYBR Green (Applied Biosystems). Primers used can be found in Table S1.

### Post-translational modification scan

RAW 264.7 cells were prepared in RPMI 1640 and infected at an MOI of 40 as described above with Δ*rgg2*, Δ*rgg3*, Δ*rgg2* + Δ*rgg3*, or bacteria-free media. Cells were collected after 30 min and lysed in a 9M urea phospho-protective lysis buffer (9 M sequanol grade urea, 20 mM HEPES, pH 8.0, 1 mM β-glycerophosphate, 1 mM sodium vanadate, and 2.5 mM sodium pyrophosphate). Protein concentration of each sample was measured using a DC Protein Assay (Bio-Rad) and a Qubit Protein Assay (Invitrogen), and their concentrations were normalized accordingly. Samples were then sent to Cell Signaling Technology for their PhosphoScan services and analysis. Proteins were digested with trypsin and then loaded to a 50 cm × 100 µm PicoFrit column packed with C18 reversed-phase resin. The column was developed with a 150-minute linear gradient in 0.125% formic acid delivered at 280 nL/min. The eluted peptides were lyophilized and then phospho-enriched by immobilized metal affinity chromatography using Fe-NTA magnetic beads (Cell Signaling Technology). Phosphorylated peptides were eluted off the beads with a basic buffer and then analyzed by tandem mass spectrometry with parameters optimized by the company. Spectra were evaluated using Comet (58) and the Core platform at Harvard University. Searches were performed against the most recent update (2021) of the Uniprot *Mus musculus* database with mass accuracy of ±5 ppm for precursor ions and 0.02 Da for product ions. Results were filtered based on mass accuracy of ±5 ppm on precursor ions and the presence of the intended motif, and then further filtered to a 1% false discovery rate.

### PTM analysis

Any measured protein that did not meet the following quality control criteria was removed before analysis: maximum abundance greater than 1,000,000, maximum coefficient of variance less than 0.5, and the number of distinct peptides that map to the protein greater than 1. PTM-SEA was then performed using genepattern.com with their default settings, comparing the data set to both the mouse and human PTM signature databases. Enrichment scores for each annotated pathway of each sample were averaged by experimental group, and their differences in means were compared using ANOVA followed by a Tukey post-hoc test.

The data set was also analyzed using Ingenuity Pathway Analysis (IPA) and by biological process gene ontology (GO) analysis using pantherdb.com. Here, only proteins that exhibited at least a 2.5-fold change between any two groups were analyzed. Default analysis settings were used for both analyses.

### Western blot

RAW 264.7 cells were prepared in RPMI 1640 and infected as described above with Δ*rgg2*, Δ*rgg3*, Δ*rgg2* + Δ*rgg3* GAS, or bacteria-free media. Selected wells were also stimulated with 100 ng/mL of LPS (Sigma). Cells were collected in 15-min intervals up to 1 h post-infection. Cells were then lysed in urea lysis buffer with 1% 2-mercaptoethanol and boiled for up to 5 min. Proteins were segregated by electrophoresis through a 4–12% Bis-Tris polyacrylamide gel (Invivogen), transferred to a PVDF membrane, and probed for IKKɑ/β, phospho-IKKɑ/β (serine 176/180), IκBɑ, and actin. Anti-rabbit or anti-mouse IgG conjugated to HRP was used as secondary antibodies. Blots were developed using SuperSignal West Femto Maximum Sensitivity Substrate (Thermo). All antibodies were purchased from Cell Signaling Technology.

### Microscopy

BMDMs were prepared as described and seeded into 8-well glass microscope slides (Millicell EZ slide, Millipore) and incubated overnight at 37°C. The following morning, the cells were infected as described with Δ*rgg2*, Δ*rgg3*, Δ*rgg2* + Δ*rgg3* GAS, or bacteria-free media at an MOI of 50. At sequential 15-minute intervals post-infection, cells were washed with PBS and fixed with 4% paraformaldehyde for 15 min. The cells were then washed three times with PBS and then blocked with 5% BSA and 0.3% Triton-X in PBS for 60 min at room temperature or 4°C overnight. The blocking buffer was then replaced with PBS containing 1% BSA, 0.3% Triton-X, and anti-p65 antibody (Cell Signaling Technology) and then incubated at 4°C overnight. Cells were then washed and incubated with Alexa Fluor 488 anti-rabbit IgG (H + L) F(ab) 2 antibody (Cell Signaling Technology) for 2 h at room temperature. 1 g/ml of DAPI (Biotium) was added for 5 min, and then, the cells were washed three times. Glass coverslips were mounted onto slides with Prolong Diamond Antifade Mountant (Invitrogen) and cured overnight at room temperature in the dark. Slides were visualized on an inverted fluorescent microscope (Keyence BZ-X710).

### NFκB translocation assays

Differentiated THP-1 cells were prepared in RPMI 1640 and infected as described above with Δ*rgg2*, Δ*rgg3*, Δ*rgg2* + Δ*rgg3* GAS, or bacteria-free media and collected after 35 min post-infection. Lysis, nuclear isolation, and the subsequent assay were performed according to kit manufacturer instructions (TransAM NFκB Family Kit from Active Motif). Nuclear protein input was normalized using a Bradford assay (Bio Rad). To determine the ratio of nuclear:cytoplasmic occupancy of p65, immunofluorescence images were used to quantify p65 fluorescence intensity. Images were converted to grayscale and were used for quantification with no other manipulations applied. A CellProfiler (Broad Institute) pipeline was followed based on the Human MCF7 and A549 cells cytoplasm–nucleus translocation (accession number BBBC014) (REF doi.org/10.1038/nmeth.2083). Briefly, following illumination corrections, each cell’s nucleus and cytoplasm were identified, and mean fluorescence intensities of p65 were quantified for both. Shown is the average ratio of intensities for each of five fields visualized (2,000 cells total) for each condition.

### Pathway inhibition and induction

Pharmacological inhibitors were added either 2.5 h prior to infection or the day before infection at the time of seeding. Inducers were added 30 min prior to infection or the day before infection at the time of seeding. Cells were treated with the agent during the infection and through the time they were collected. Concentrations were as follows: 20 µM AKT1/2 inhibitor (Sigma A6730); 7.5 µM TWS119 (Sigma); 7.5 µM DIF-3 (MedChem Express); 10 µM SC79 (MedChem Express); 2 µM CDK2 inhibitor II (Cayman); 5 µM GSK126 (Selleck Chemicals); 500 nM Suberoylanilide hydroxamic acid (SAHA); 10 µM EX527 (Cayman); 5 µM AGK2 (Cayman); and 1 µM BRM014 (Cayman). Concentrations were chosen by those commonly found in the literature and titrations to determine a maximum concentration without disrupting other normal cellular processes.

### NFκB reporter assay

35,000 RAW Blue cells were plated in a 96-well plate the day before infection. The following day, cells were infected with GAS at an MOI of 10 as described above. One hundred fifty microliters of Quanti-Blue Reagent (Invivogen) were mixed with 50 µL of sample supernatant in a new 96-well plate, and the absorbance was read at 625 nm every 30 min for 6 h.

### Reinfections

For primary infections, Raw-Blue cells were seeded and infected as described above in 6-well plates, and gentamycin was kept in the medium for 20 h. The day after the infection (20.5 h.p.i.), fresh medium without antibiotic was used to wash cells. Cells were subsequently infected with GAS bacteria for 30 min with subsequent addition of antibiotics and further incubation for 20 h. Culture supernatants were collected, and NFκB activity was determined by Quanti-Blue assay.

### ELISA

RAW 264.7 or THP-1 cells were prepared and infected as described above. At appropriate time points described in the text, supernatants were collected, centrifuged, re-collected, and frozen at −80°C. All ELISAs were performed with kits provided by BioLegend.

## Data Availability

The raw and processed data from the PTM scan can be found in the MassIVE repository. doi:10.25345/C51834F1P. Raw RNAseq data is available on Gene Expression Omnibus, reference GSE289076.

## SUPPLEMENTAL MATERIAL

Data set S1 mbio.00373-25-s0001.csv Macrophage RNAseq and differential expression between uninfected, QS-OFF, QS-ON, and mixed ON/OFF.

Data set S2 mbio.00373-25-s0002.csv Macrophage phosphoproteomic reads comparing uninfected, QS-OFF, and QS-ON.

Supplemental material mbio.00373-25-s0003.docx Fig. S1 to S4; Tables S1 and S2.

ASM does not own the copyrights to Supplemental Material that may be linked to, or accessed through, an article. The authors have granted ASM a non-exclusive, world-wide license to publish the Supplemental Material files. Please contact the corresponding author directly for reuse.
