# Supplementary material for: The Streptococcus pyogenes Rgg2/Rgg3 quorum sensing system causes global suppression of macrophage inflammatory programs via an intranuclear mechanism
Source: mBio. 2025 Sep 25;16(11):e00373-25. doi: 10.1128/mbio.00373-25 (PMC12607563; doi:10.1128/mbio.00373-25)
Supplement: Supplemental material — Fig. S1 to S4; Tables S1 and S2. [file mbio.00373-25-s0003.docx]

**Supplemental information**

**The *Streptococcus pyogenes* Rgg2/Rgg3 quorum sensing system causes global suppression of macrophage inflammatory programs via an intranuclear mechanism**

**Authors**: Sam F. Feldstein, Kate M. Rahbari, Trevor R. Leonardo, Suzanne A. Alvernaz, Ian McIntire, Richard Foster, Michael J. Federle

**Contents**

Figure S1……………………………………..Page 2

Figure S2……………………………………..Page 3

Figure S3a……………………………………Page 4

Figure S3b……………………………………Page 5

Figure S3c……………………………………Page 6

Figure S4……………………………………..Page 7

Table S1……….……………………………..Page 8

Table S2: Cluster V genes (Fig. 2)…….Page 9

**Data tables available as separate files:**

Dataset S1: RNA-seq counts of infected macrophages.

Dataset S2: Phosphoproteome counts of infected macrophages.

**
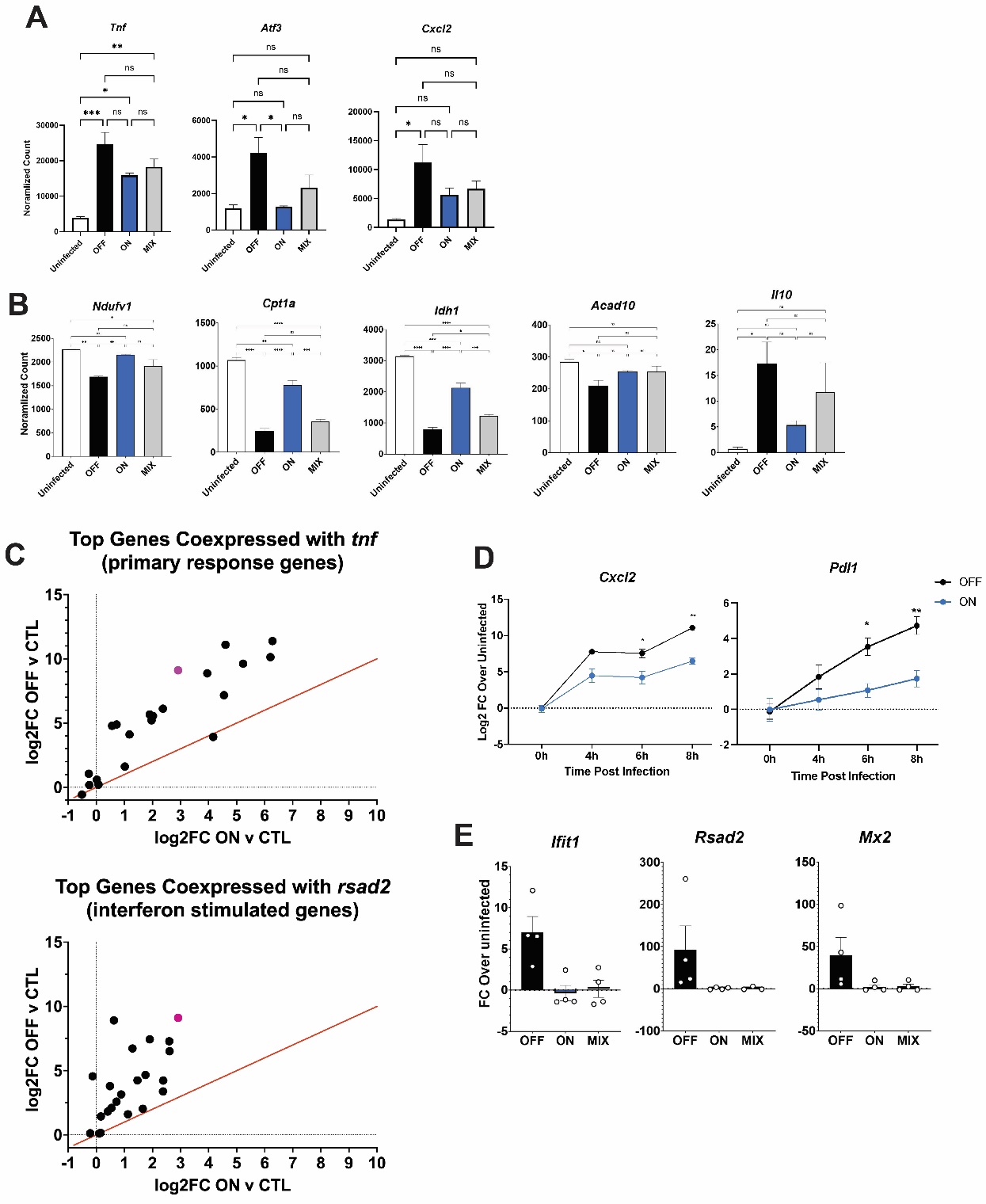
**

**Figure S1.**

1. Normalized read counts of infected RAW264.7 cells at 2h post-infection (p.i.) of select early response genes from RNA-seq.
2. Normalized read counts at 8h p.i. from RNA-seq dataset of select genes associated with oxidative phosphorylation and fatty acid metabolism, and *Il10.*
3. Comparison of log_2_ fold changes of OFF-infected over uninfected samples, and to ON-infected over uninfected samples, averaged across all time points from RNA-seq dataset for genes most commonly co-expressed with *Tnf* and *Rsad2*. Genes most commonly co-expressed with these genes was determined using ARCHS4’s RNA-seq gene-gene co-expression matrix acessed via Enrichr. Orange line shows line of identity.
4. Time course of gene expression for *Cxcl2* and *Pdl1* for OFF and ON-infected RAW264.7 cells.
5. RT-qPCR of select type I interferon stimulated genes showing fold change over uninfected cells at 4h p.i.

**Figure S2.** Enrichment of gene ontology (GO) biological processes clusters I-IV (A-D, respectively) created from analysis of RNA-seq dataset at 8h post-infection.

**
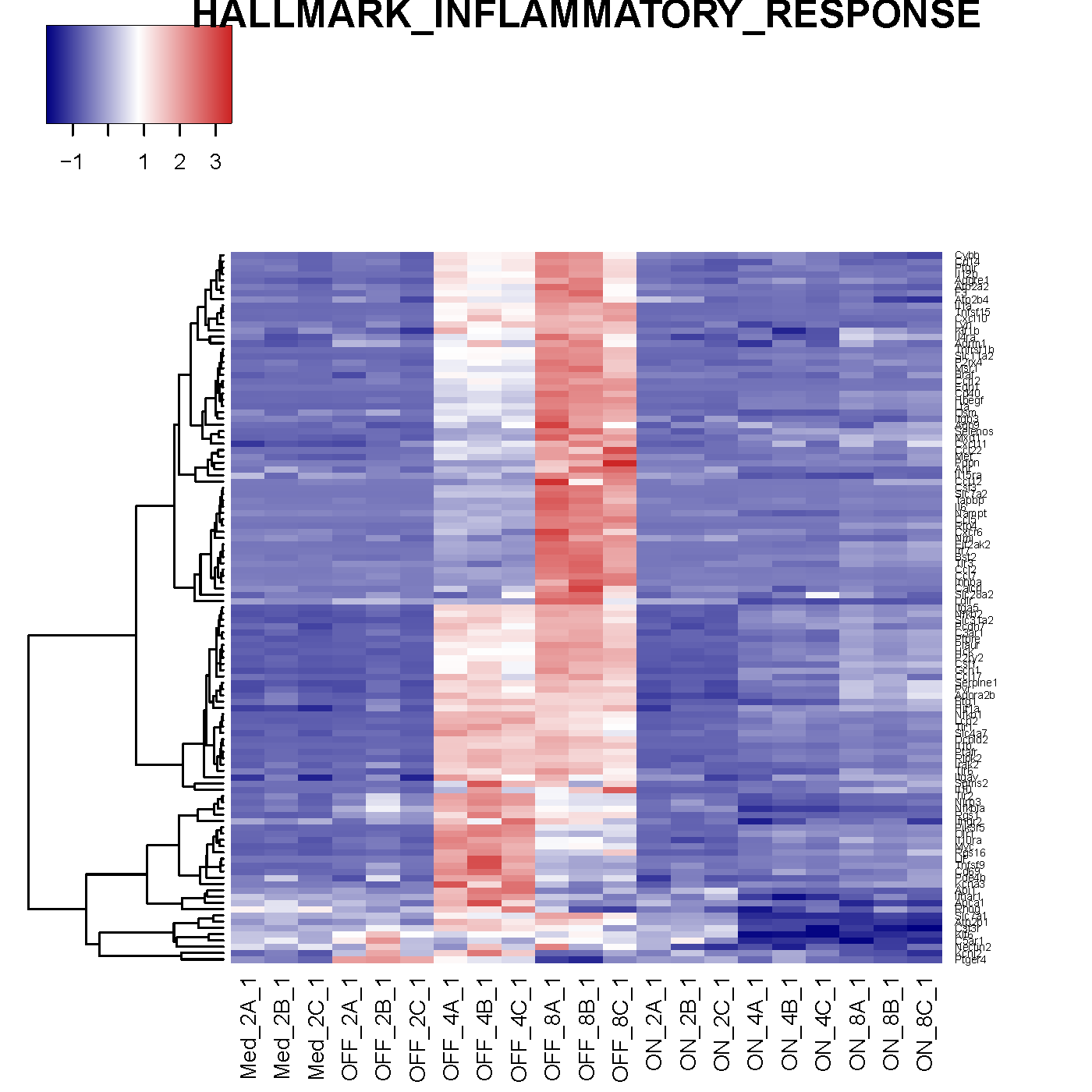
**

**Figure S3a: GSEA Hallmark_Inflammatory_Reseponse heat map (used for Figure 1C)**

**
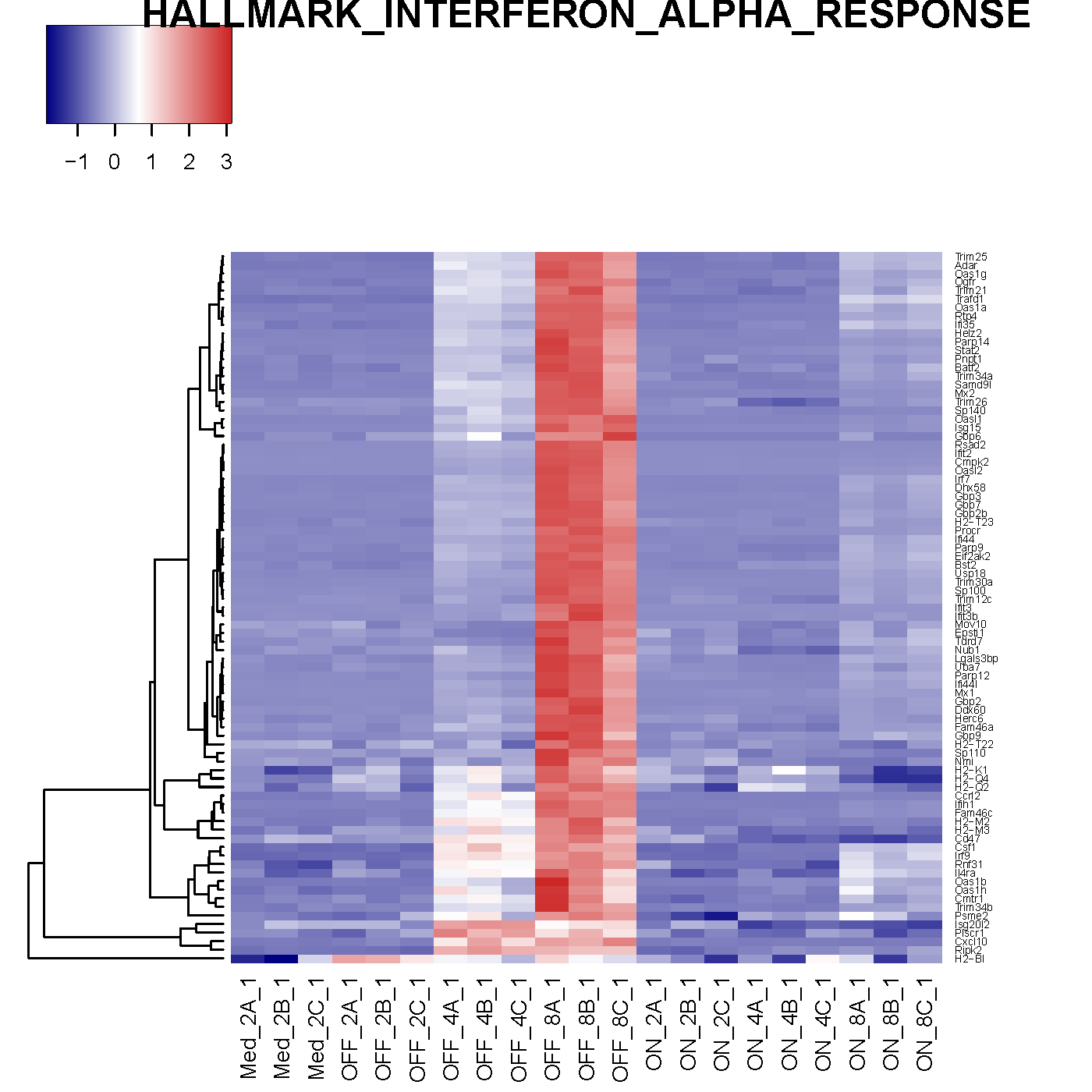
**

**Figure S3b: GSEA Hallmark_IFNa_Signaling heat map (used for Figure 1C).**

**
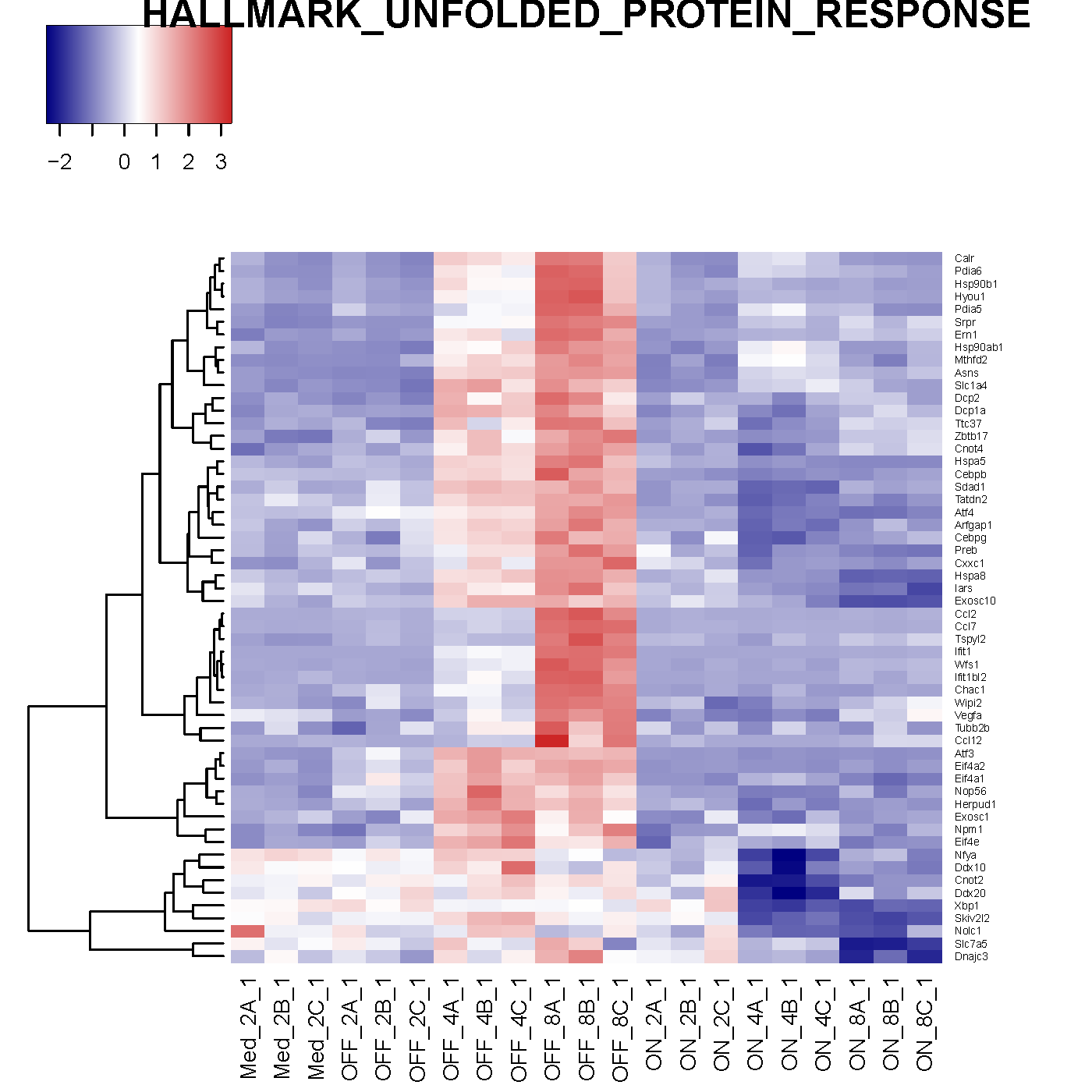
**

**Figure S3c: GSEA Hallmark_Unfolded_Protein_Respons heat map (used from Figure 1C).**

**
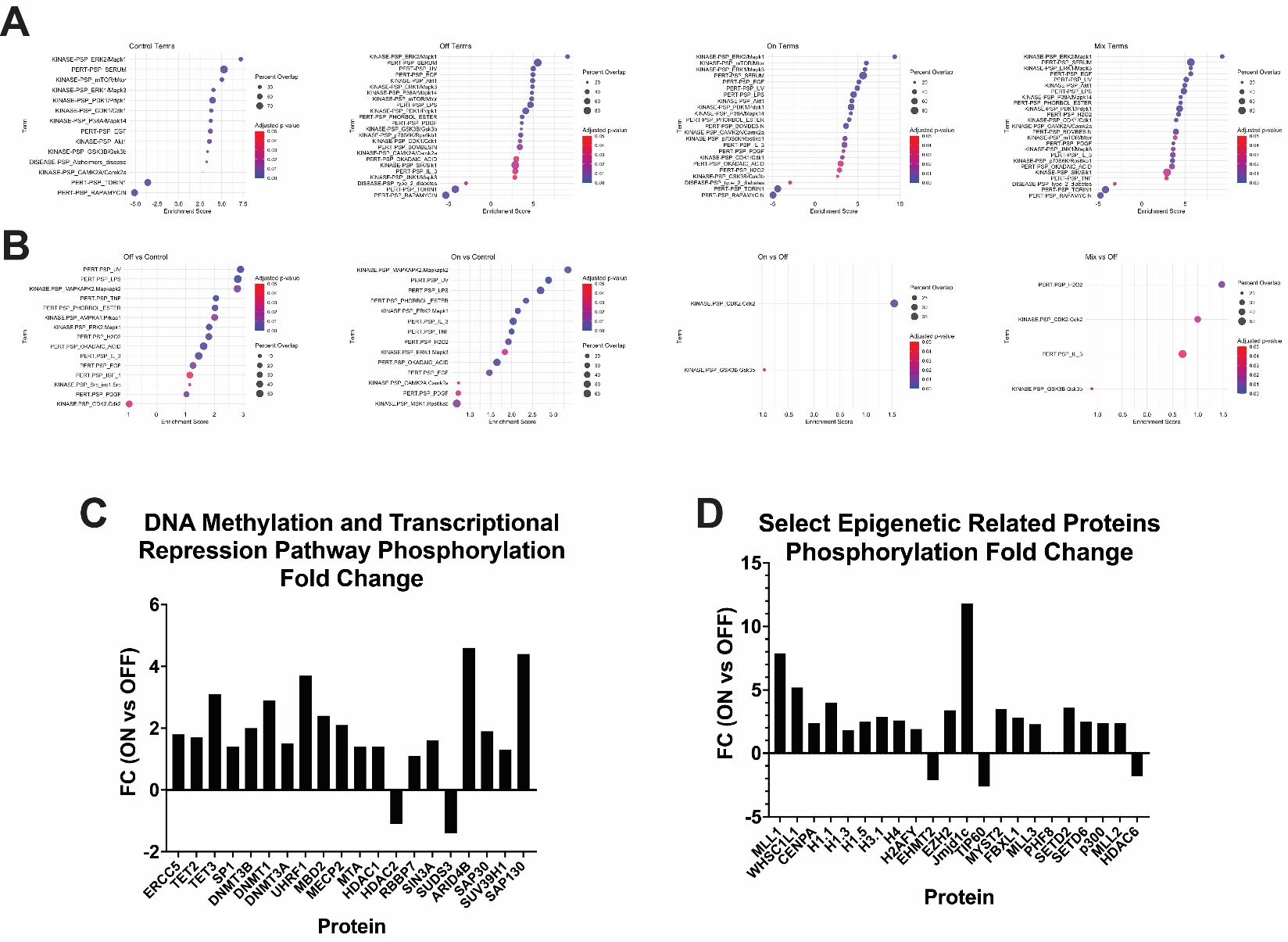
**

**Figure S4.**

1. PTM-SEA pathways that are significantly enriched (fdr-adjusted p-value<.05) from the post translational modification scan of each infection condition
2. PTM-SEA pathways that show significant differences in enrichments scores (p-value<0.05) in select pairwise comparisons. Significance determined by one way ANOVA.
3. Differential phosphorylation of DNA methylation and Transcriptional Repression pathway proteins.
4. Differential phosphorylation of Epigenetic Related pathway proteins.

**Supplemental Table 1. Primers used for RT-qPCR.**

| **Primer** | **Sequence** |
| --- | --- |
| *gnl1*_F (housekeeping) | GCGGAAGCGAGGGCT |
| *gnl1*_R (housekeeping) | CCTGGGAAGGTTGCTGGTT |
| *tnf*_F | TGGAACTGGCAGAAGAGGCAC |
| *tnf*_R | TAGAGGCTGAGACATAGGCACCG |
| *rsad2*_F | CCCCGTGAGTGTCAACTACC |
| *rsad2*_R | TCTTCTCCAAACCAGCCTGT |
| *ifit1*_F | CCAAGTGTTCCAATGCTCCT |
| *ifit1*_R | GGATGGAATTGCCTGCTAGA |
| *mx2*_F | GTGGCAGAGGGAGAATGTCG |
| *mx2*_R | CTCGTCCACGGTACTGCTTT |
| *nos2*_F | GTTCTCAGCCCAACAATACAAGA |
| *nos2*_R | GTGGACGGGTCGATGTCAC |
| *cxcl2*_F | TCCAAAAGATACTGAACAAAGGCA |
| *cxcl2*_R | GCACATCAGGTACGATCCAG |
| *arg1*_F | ATTGGCTTGCGAGACGTAGA |
| *arg1*_R | GGCCTTTTCTTCCTTCCCAG |
| *klf4*_F | GTGCCCCGACTAACCGTTG |
| *klf4*_R | GTCGTTGAACTCCTCGGTCT |

**Supplemental Table S2: Cluster V genes from Figure 2.**

| **gene** | **ON vs OFF log2FC** | **padj** | **ON vs CTL log2FC** | **padj2** |
| --- | --- | --- | --- | --- |
| **Sept4** | 2.95059636 | 3.08E-05 | 3.598503 | 2.59E-02 |
| **1600010M07Rik** | 1.498752862 | 8.28E-06 | 1.487019 | 2.82E-02 |
| **1700001P01Rik** | 1.881495864 | 0.012446771 | 3.495256 | 2.35E-02 |
| **1700016G22Rik** | 3.315352644 | 0.007209107 | 5.938816 | 3.34E-02 |
| **1700056E22Rik** | 1.829627884 | 0.039502177 | 5.308651 | 2.42E-02 |
| **1700110C19Rik** | 1.991931376 | 0.022405492 | 5.848354 | 1.41E-02 |
| **2810454H06Rik** | 2.60377953 | 2.35E-05 | 3.163143 | 1.88E-02 |
| **3830432H09Rik** | 2.705769356 | 0.001830171 | 5.387243 | 2.47E-02 |
| **4833418N02Rik** | 2.203564648 | 9.85E-06 | 2.063325 | 3.77E-02 |
| **4933433G15Rik** | 2.187336608 | 7.37E-05 | 3.49974 | 7.00E-03 |
| **9230114K14Rik** | 2.376225499 | 1.65E-05 | 2.608311 | 1.70E-02 |
| **9530053A07Rik** | 3.128890166 | 0.018597551 | 7.398189 | 1.24E-02 |
| **A930001C03Rik** | 2.419941722 | 0.013232423 | 5.017402 | 3.56E-02 |
| **Abcg4** | 1.686622878 | 0.030651497 | 3.633256 | 2.33E-02 |
| **Ager** | 1.488195107 | 0.004439172 | 2.274069 | 2.54E-02 |
| **Ak1** | 2.109126846 | 0.000194424 | 2.413496 | 2.86E-02 |
| **Amh** | 1.628070596 | 0.003409334 | 3.69085 | 1.03E-03 |
| **Ampd1** | 2.94882042 | 0.009198877 | 4.599351 | 4.80E-02 |
| **Amt** | 1.372968366 | 0.002942064 | 2.027828 | 2.52E-02 |
| **Ankdd1b** | 2.947193382 | 0.008321342 | 5.494373 | 3.56E-02 |
| **Ankrd6** | 3.610872955 | 0.000190081 | 7.210372 | 3.52E-03 |
| **Aoc3** | 1.765164854 | 0.028301208 | 4.755477 | 6.25E-03 |
| **Ap3m2** | 0.368042743 | 0.033241821 | 0.710076 | 2.91E-02 |
| **Apoe** | 0.34704225 | 0.03582044 | 0.797962 | 7.65E-03 |
| **Arid3c** | 3.52075207 | 0.003803711 | 5.285679 | 4.76E-02 |
| **Arl4d** | 3.185967232 | 6.77E-11 | 2.515175 | 7.74E-03 |
| **Azin2** | 1.148281127 | 0.000202245 | 1.511943 | 1.64E-02 |
| **B130034C11Rik** | 0.921450826 | 0.004993409 | 1.3907 | 3.11E-02 |
| **Baiap3** | 3.481521115 | 0.010462435 | 7.826793 | 1.18E-02 |
| **Best2** | 2.912488817 | 0.000632289 | 4.55094 | 3.66E-02 |
| **Bspry** | 2.379918948 | 0.002016448 | 3.320828 | 3.70E-02 |
| **C130036L24Rik** | 2.20643451 | 7.98E-06 | 2.583772 | 7.00E-03 |
| **C130083M11Rik** | 1.929867281 | 3.12E-05 | 2.077007 | 2.35E-02 |
| **Camsap3** | 2.01220259 | 0.002391685 | 3.633791 | 3.52E-02 |
| **Ccdc39** | 0.881333537 | 0.002306892 | 1.443826 | 1.38E-02 |
| **Ccno** | 3.847345652 | 1.90E-07 | 3.52733 | 1.70E-02 |
| **Cd79a** | 3.990711464 | 3.39E-06 | 4.23459 | 2.09E-02 |
| **Cdc42bpg** | 1.218027256 | 0.004937134 | 1.772408 | 3.13E-02 |
| **Cdh24** | 1.537848055 | 0.009845784 | 2.754522 | 1.58E-02 |
| **Celf5** | 4.687759185 | 2.96E-05 | 5.147253 | 3.73E-02 |
| **Col7a1** | 1.685729914 | 0.000756729 | 1.986859 | 4.47E-02 |
| **Colec11** | 5.406765282 | 3.46E-05 | 6.164092 | 2.54E-02 |
| **Cpt1c** | 2.654352238 | 1.16E-05 | 2.938242 | 1.70E-02 |
| **Crip2** | 3.422265269 | 0.000103339 | 4.452231 | 9.99E-03 |
| **Crip3** | 3.535689897 | 0.013656345 | 6.874202 | 3.09E-02 |
| **Cux2** | 2.431369551 | 0.000267024 | 3.218628 | 2.10E-02 |
| **Cuzd1** | 2.695218338 | 0.026896054 | 6.144354 | 3.06E-02 |
| **Cyp27b1** | 1.536718989 | 0.007779878 | 2.331756 | 3.98E-02 |
| **Cyp2t4** | 3.172193536 | 1.19E-06 | 2.958851 | 2.53E-02 |
| **Daam2** | 2.019316384 | 9.64E-07 | 1.769502 | 3.10E-02 |
| **Dll3** | 2.021964023 | 0.019019451 | 4.794172 | 4.77E-02 |
| **Dusp15** | 1.532554029 | 0.041749702 | 5.22958 | 1.81E-02 |
| **E230025N22Rik** | 1.006726022 | 0.037500506 | 2.454939 | 1.42E-02 |
| **Efnb2** | 2.561161784 | 0.000143057 | 2.757858 | 4.28E-02 |
| **Epha2** | 1.332370623 | 0.015291409 | 2.103442 | 4.35E-02 |
| **Etv2** | 5.453453563 | 1.33E-07 | 5.569597 | 1.62E-02 |
| **Evpl** | 2.317051548 | 0.003823333 | 6.827337 | 3.44E-03 |
| **F2rl3** | 2.251094866 | 0.012218494 | 5.04384 | 2.91E-02 |
| **Fam198b** | 1.900817199 | 0.003260132 | 2.715217 | 2.82E-02 |
| **Fam43a** | 1.614294889 | 0.008040043 | 3.047899 | 1.88E-02 |
| **Fbxw9** | 0.787439638 | 0.007271837 | 1.19725 | 3.13E-02 |
| **Fndc8** | 1.44957 | 0.006722973 | 3.218844 | 6.21E-03 |
| **Foxd4** | 3.240779841 | 0.012017479 | 5.734935 | 4.99E-02 |
| **Fscn1** | 1.466547689 | 0.012275751 | 2.943754 | 1.00E-02 |
| **G6b** | 4.153296107 | 8.61E-05 | 5.838814 | 2.38E-02 |
| **Gdpd2** | 2.10738375 | 0.004922071 | 4.843564 | 2.20E-02 |
| **Gfap** | 2.124935329 | 4.30E-05 | 3.160914 | 5.64E-03 |
| **Gm10433** | 3.559165907 | 0.000142715 | 6.551803 | 6.25E-03 |
| **Gm15441** | 1.614099506 | 5.40E-05 | 1.702302 | 3.56E-02 |
| **Gm15545** | 1.014317761 | 0.000100429 | 1.151238 | 2.49E-02 |
| **Gm16486** | 3.795961343 | 0.001764129 | 5.841714 | 3.06E-02 |
| **Gnas** | 0.422925868 | 0.00817427 | 0.627448 | 3.98E-02 |
| **Gnmt** | 1.813242385 | 0.000148765 | 2.746652 | 1.39E-02 |
| **Gp5** | 2.645036297 | 0.001869744 | 5.72208 | 9.99E-03 |
| **Gpr182** | 2.258752328 | 0.000861685 | 2.774387 | 4.84E-02 |
| **Gpt** | 0.536754468 | 0.005261362 | 1.034303 | 5.64E-03 |
| **Grhl3** | 3.173533419 | 0.003065675 | 6.065234 | 1.99E-02 |
| **Gstp3** | 2.784177957 | 0.037125748 | 7.40014 | 1.62E-02 |
| **Gtf2a2** | 0.268481128 | 0.00230625 | 0.357144 | 3.53E-02 |
| **Gxylt2** | 3.690011418 | 0.003647153 | 6.19507 | 3.06E-02 |
| **Gzmm** | 1.923744878 | 7.57E-05 | 2.342472 | 1.58E-02 |
| **Hexim1** | 0.685496759 | 0.002515162 | 0.87359 | 4.87E-02 |
| **Hist2h4** | 2.773597614 | 0.000679551 | 4.505241 | 2.24E-02 |
| **Hnf1a** | 2.014671828 | 0.046997973 | 6.111403 | 1.70E-02 |
| **Hypk** | 0.827065214 | 1.35E-05 | 1.012285 | 6.46E-03 |
| **Icam5** | 1.470330099 | 0.014335112 | 2.466682 | 3.45E-02 |
| **Il17rb** | 2.067154796 | 0.02936338 | 5.329239 | 2.81E-02 |
| **Iqcf1** | 2.002603712 | 0.002286176 | 4.786509 | 1.74E-02 |
| **Itga10** | 0.898851146 | 0.023503629 | 1.595246 | 3.34E-02 |
| **Jsrp1** | 2.061019625 | 0.013075933 | 3.901877 | 2.43E-02 |
| **Kcnd3** | 1.69728193 | 0.049445775 | 5.338305 | 2.21E-02 |
| **Kdelr3** | 1.955009857 | 0.000707133 | 2.569409 | 2.82E-02 |
| **Lama5** | 2.105051689 | 0.003166783 | 3.941098 | 2.89E-02 |
| **Ldb3** | 2.659916279 | 0.002110078 | 3.94424 | 4.47E-02 |
| **Lhx3** | 5.257038151 | 4.52E-05 | 6.664509 | 1.53E-02 |
| **LOC108167825** | 1.800148545 | 0.04197068 | 4.171341 | 2.87E-02 |
| **Ltb4r2** | 2.823994995 | 0.001740109 | 4.881845 | 2.21E-02 |
| **Lurap1** | 0.85718433 | 0.008233179 | 1.266312 | 4.98E-02 |
| **Mab21l3** | 3.83645695 | 5.39E-05 | 6.057535 | 1.48E-03 |
| **Map3k13** | 1.113962112 | 0.022234873 | 2.714466 | 2.08E-02 |
| **Matn1** | 3.41367797 | 0.000579178 | 6.451534 | 3.52E-03 |
| **Mc5r** | 4.13417793 | 0.002256118 | 6.216773 | 3.49E-02 |
| **Mefv** | 3.886819098 | 0.001790903 | 6.506565 | 2.20E-02 |
| **Mmel1** | 4.310955236 | 0.000809188 | 6.506833 | 2.24E-02 |
| **Mybl1** | 1.359065716 | 0.002091953 | 1.722669 | 4.51E-02 |
| **Myo1a** | 3.331177968 | 0.000245467 | 4.405701 | 3.28E-02 |
| **Nlgn3** | 2.356732504 | 0.0052755 | 6.28138 | 6.95E-03 |
| **Notch4** | 0.717337516 | 0.014313575 | 1.269033 | 3.14E-02 |
| **Npb** | 1.118073511 | 0.020307385 | 2.131652 | 3.66E-02 |
| **Nudt17** | 1.151860869 | 5.31E-05 | 1.225075 | 3.07E-02 |
| **Nudt8** | 1.49295994 | 0.004874111 | 2.30236 | 2.52E-02 |
| **Nup210l** | 1.30789589 | 0.005710363 | 2.118829 | 2.10E-02 |
| **Ovgp1** | 2.558663728 | 0.000166749 | 3.396302 | 1.08E-02 |
| **Pdrg1** | 0.855845585 | 0.020828543 | 1.460704 | 3.52E-02 |
| **Pdzd4** | 1.179871891 | 0.038186963 | 2.778852 | 1.85E-02 |
| **Pfkfb2** | 0.411650703 | 6.69E-05 | 0.477382 | 1.88E-02 |
| **Phkg1** | 3.433188506 | 4.59E-06 | 5.140071 | 2.85E-03 |
| **Phlda3** | 1.521858963 | 0.021547677 | 2.730987 | 2.64E-02 |
| **Phyhip** | 4.681352922 | 0.004004361 | 7.021218 | 4.33E-02 |
| **Pip5kl1** | 2.60092178 | 0.00839736 | 4.588252 | 2.86E-02 |
| **Pkd1l3** | 1.245875046 | 0.003820771 | 1.836066 | 3.09E-02 |
| **Pla2g4f** | 3.34576596 | 2.17E-06 | 4.20769 | 1.53E-02 |
| **Plek2** | 2.645162106 | 0.010563606 | 6.177647 | 2.02E-02 |
| **Plekhg4** | 1.022407934 | 0.047195421 | 2.976273 | 3.00E-03 |
| **Popdc2** | 2.699549169 | 0.001082799 | 4.69312 | 1.35E-02 |
| **Ppp1r3f** | 1.073077559 | 0.007110686 | 1.778706 | 3.63E-02 |
| **Psrc1** | 1.195080197 | 0.00044956 | 1.445985 | 2.82E-02 |
| **Rab30** | 1.907720869 | 0.025899021 | 5.090194 | 3.37E-02 |
| **Rem2** | 2.7948063 | 0.000978268 | 3.761616 | 4.87E-02 |
| **Rin1** | 1.57343762 | 3.87E-14 | 1.558163 | 1.17E-04 |
| **Rnf183** | 0.881217917 | 0.011025074 | 1.422779 | 3.49E-02 |
| **Rnf39** | 3.287175331 | 0.000132318 | 3.727686 | 3.74E-02 |
| **Robo3** | 4.23975015 | 0.000742029 | 7.055808 | 1.38E-02 |
| **Rps6kl1** | 1.074458444 | 3.69E-06 | 0.949475 | 4.16E-02 |
| **S1pr4** | 3.13973129 | 1.37E-05 | 3.523269 | 1.70E-02 |
| **Sdk1** | 2.4824605 | 0.001883936 | 5.335007 | 5.73E-03 |
| **Serinc2** | 0.670125779 | 0.011183455 | 1.038757 | 4.20E-02 |
| **Sh2d5** | 1.251741334 | 0.005358476 | 2.168144 | 1.38E-02 |
| **Shd** | 1.940315806 | 0.010464052 | 3.340418 | 2.99E-02 |
| **Slc16a8** | 1.141281427 | 0.000356946 | 1.742356 | 8.45E-03 |
| **Slc19a2** | 1.287933506 | 0.000534321 | 1.490348 | 3.99E-02 |
| **Slc23a3** | 2.684031819 | 0.001209842 | 4.881912 | 1.58E-02 |
| **Slc35a1** | 0.348617488 | 1.85E-06 | 0.363717 | 1.16E-02 |
| **Slc5a5** | 2.46402905 | 0.002581312 | 3.960731 | 2.08E-02 |
| **Smim24** | 2.822798613 | 0.005496727 | 5.719655 | 1.38E-02 |
| **Stab1** | 0.73460571 | 0.022234873 | 1.275555 | 3.37E-02 |
| **Sv2a** | 3.143018251 | 6.71E-07 | 3.329519 | 7.74E-03 |
| **Syngr3** | 3.664487513 | 4.30E-05 | 5.19096 | 1.38E-02 |
| **Syngr4** | 1.464797753 | 0.00080361 | 2.14944 | 3.13E-02 |
| **Synpo2l** | 2.723863319 | 7.82E-05 | 3.237234 | 2.45E-02 |
| **Tada3** | 0.564760966 | 6.61E-08 | 0.481489 | 2.10E-02 |
| **Tcta** | 0.822586371 | 0.045171049 | 1.599934 | 3.66E-02 |
| **Tctex1d4** | 0.810122524 | 0.020632452 | 1.6261 | 2.18E-02 |
| **Tctn1** | 0.859161529 | 0.000454619 | 1.090406 | 2.20E-02 |
| **Tlcd2** | 2.643381416 | 0.000308389 | 4.38301 | 1.81E-03 |
| **Tmc4** | 1.080859283 | 0.000307399 | 2.020126 | 9.91E-04 |
| **Tmem44** | 3.281511847 | 0.000116253 | 4.705236 | 3.20E-02 |
| **Tmem79** | 2.101068645 | 7.64E-06 | 2.071819 | 2.51E-02 |
| **Tnni1** | 2.761939235 | 0.023771554 | 5.858589 | 3.77E-02 |
| **Trim72** | 3.640391214 | 4.09E-05 | 4.299029 | 2.33E-02 |
| **Trp53inp1** | 2.025252883 | 0.007767689 | 3.472923 | 1.58E-02 |
| **Tspoap1** | 1.263561271 | 0.00217344 | 1.932765 | 1.42E-02 |
| **Tssk3** | 3.368943639 | 3.54E-05 | 4.236568 | 2.45E-02 |
| **Tssk4** | 1.543743059 | 0.002992958 | 2.378693 | 2.25E-02 |
| **Ttll9** | 2.309593895 | 0.007173619 | 6.478888 | 6.21E-03 |
| **Tubb1** | 2.795588289 | 0.006315836 | 6.790318 | 8.45E-03 |
| **Unc13d** | 1.341144418 | 0.014658268 | 2.418547 | 3.56E-02 |
| **Ushbp1** | 3.543652811 | 0.000115414 | 3.986635 | 4.38E-02 |
| **Vwa1** | 1.617206813 | 0.000335595 | 2.018672 | 3.20E-02 |
| **Vwa5b2** | 3.304373774 | 0.000148645 | 4.081851 | 3.34E-02 |
| **Vwa7** | 0.93077556 | 0.000185221 | 1.293579 | 9.99E-03 |
| **Wdr78** | 0.73825701 | 0.007528358 | 1.195697 | 3.09E-02 |
| **Wnk4** | 1.977899939 | 0.000614576 | 3.557242 | 1.44E-03 |
| **Zfp213** | 0.475148033 | 0.046057652 | 0.992817 | 2.66E-02 |
| **Zfp365** | 4.618207106 | 4.91E-05 | 6.952409 | 1.27E-02 |
| **Zfp36l3** | 0.998381107 | 0.034072702 | 2.349167 | 1.70E-02 |
| **Zswim3** | 0.562985817 | 0.047642226 | 1.126008 | 3.20E-02 |
